# Cancer stem cell–immune cell crosstalk in breast tumor microenvironment: a determinant of therapeutic facet

**DOI:** 10.3389/fimmu.2023.1245421

**Published:** 2023-11-27

**Authors:** Aishwarya Guha, Kuntal Kanti Goswami, Jasmine Sultana, Nilanjan Ganguly, Pritha Roy Choudhury, Mohona Chakravarti, Avishek Bhuniya, Anirban Sarkar, Saurav Bera, Sukanya Dhar, Juhina Das, Tapasi Das, Rathindranath Baral, Anamika Bose, Saptak Banerjee

**Affiliations:** ^1^ Department of Immunoregulation and Immunodiagnostics, Chittaranjan National Cancer Institute, Kolkata, India; ^2^ Department of Microbiology, Asutosh College, Kolkata, India; ^3^ Department of Pharmaceutical Technology Biotechnology National Institute of Pharmaceutical Education and Research (NIPER) Sahibzada Ajit Singh (S.A.S.) Nagar, Mohali, Punjab, India

**Keywords:** breast cancer (BC), breast cancer stem cells (BCSCs), tumor microenvironment (TME), innate immune cells, adaptive immune cells

## Abstract

Breast cancer (BC) is globally one of the leading killers among women. Within a breast tumor, a minor population of transformed cells accountable for drug resistance, survival, and metastasis is known as breast cancer stem cells (BCSCs). Several experimental lines of evidence have indicated that BCSCs influence the functionality of immune cells. They evade immune surveillance by altering the characteristics of immune cells and modulate the tumor landscape to an immune-suppressive type. They are proficient in switching from a quiescent phase (slowly cycling) to an actively proliferating phenotype with a high degree of plasticity. This review confers the relevance and impact of crosstalk between immune cells and BCSCs as a fate determinant for BC prognosis. It also focuses on current strategies for targeting these aberrant BCSCs that could open avenues for the treatment of breast carcinoma.

## 1 Introduction

The incidence as well as the mortality of breast cancer (BC) is ever increasing with significant variation among different countries. In developing countries, the BC-based mortality rate is moderately higher due to limited treatment regimens and late-stage diagnosis. BC occurs mainly due to somatic, genetic, and epigenetic alterations in a lifetime (i.e., non-hereditary), while only 5%–10% of BC cases are hereditary [majority detected with mutations in two tumor-suppressor genes: breast cancer gene 1 (*BRCA1*) and breast cancer gene 2 (*BRCA2*)]. For BC patients, the 5-year survival rate is approximately 99% for localized BC, 86% for regional diseases, and 27% for metastatic BC (stage IV).

**Figure 1:**
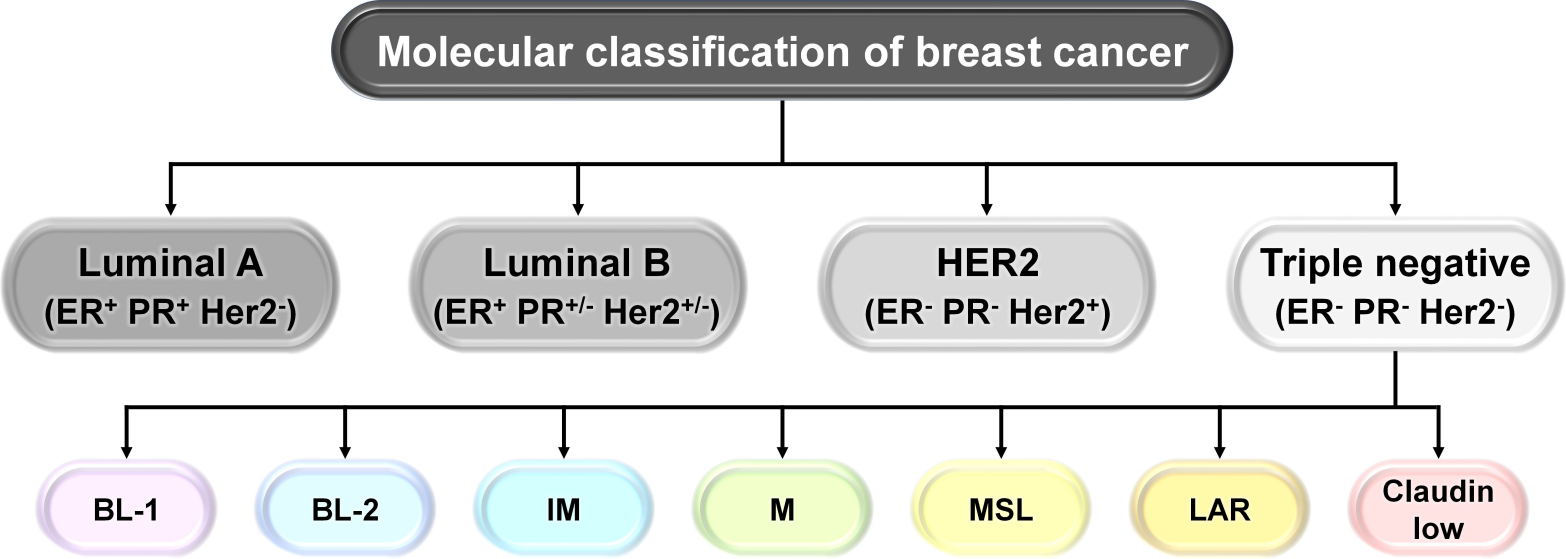
Molecular classification of breast cancer (BC). Schematic representation of the molecular classification of breast cancer: based on the presence or absence of estrogen receptor (ER), progesterone receptor (PR), and human epidermal growth factor receptor 2 (HER2), human breast carcinoma has been categorized into four different types: luminal A (ER^+^, PR^+^, HER2^−^), luminal B (ER^+^, PR^+/−^, HER2^+/−^), HER2^+^, and triple negative breast cancer (TNBC) (ER^−^, PR^−^, HER2^−^). TNBCs are further subdivided into transcriptome-based subtypes: basal cell-like type 1 (BL-1), basal cell-like type 2 (BL-2), immune-modulatory (IM), mesenchymal-like (M), mesenchymal stem cell-like (MSL), luminal androgen receptor (LAR), and claudin low.

BC represents a complex and heterogeneous microenvironment with distinct subtypes, identified through hormonal gene expression profiling. There are four molecular subgroups: luminal A (estrogen receptor, ER^+^; progesterone, PR^+^; HER2^−^), luminal B (estrogen receptor, ER^+^; progesterone, PR^+/−^; HER2^+/−^), human epidermal growth factor receptor 2 (HER2)-positive (the HER2 protein helps breast cancer cells grow quickly), and triple-negative breast cancer (TNBC) (estrogen receptor, ER^−^; progesterone receptor, PR^−^; HER2^−^) (**Figure 1**). Presently, TNBCs are further divided into the following transcriptome-based subtypes: basal cell-like type 1 (BL-1), basal cell-like type 2 (BL-2), immune-modulatory (IM), mesenchymal-like (M), mesenchymal stem cell-like (MSL), luminal androgen receptor (LAR), and claudin low (1, 2). Luminal A and luminal B subtypes, due to the presence of hormone receptors, respond to anti-estrogen or anti-progesterone therapies. TNBC, on the other hand, due to the absence of hormone receptors, is difficult to target and is considered as the most aggressive subtype of breast cancer (1, 2).

**Figure 2:**
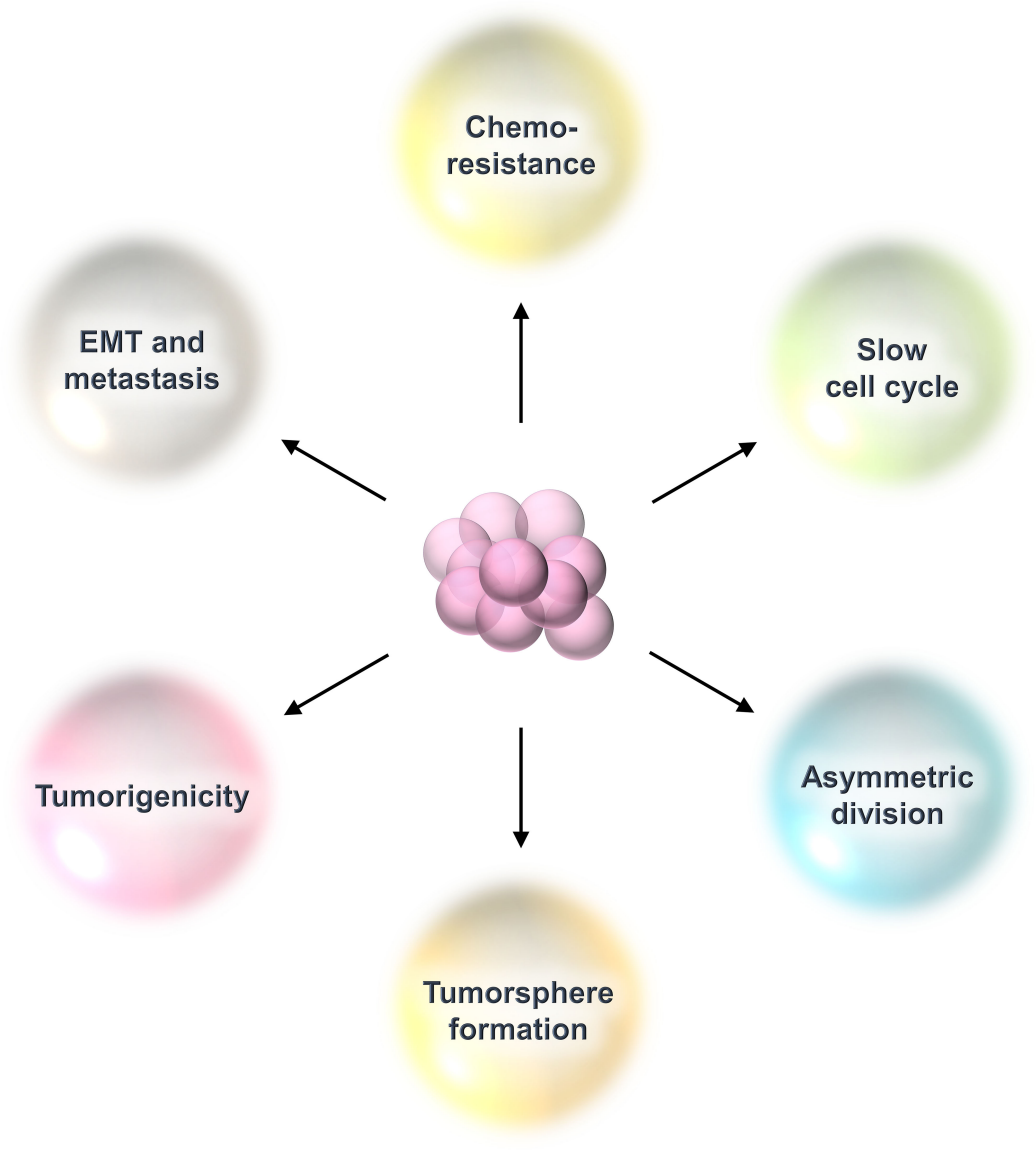
Properties of breast cancer stem cells (BCSCs). BCSCs show characteristics resembling those of typical stem cells. They are dormant cells that divide slowly. They have the capacity for self-renewal, thereby maintaining their population of undifferentiated cells. They divide asymmetrically to create daughter cells that undergo differentiation. This kind of cell division allows them to maintain their own pool while also producing the bulk of the tumor. They are immortal cells because they can withstand chemotherapy or radiation treatment. Following that, these therapy-resistant cells display all of EMT’s characteristics and a heightened ability for metastasis. They can grow in poor adherence cell culture plates under *in-vitro* conditions to form tumorspheres.

Breast cancer stem cells (BCSCs) are a rare population of undifferentiated cells that reside within the tumor and exhibit properties similar to normal stem cells and are referred to as tumor-initiating cells (3). They possess the capacity for self-renewal, which refers to a cell’s ability to divide endlessly in an undifferentiated condition (3). They replicate seldom or slowly and have infinite potential for proliferation (3). They can divide asymmetrically to produce daughter cells with the capacity to differentiate (3). Clinically, BCSCs are responsible for treatment resistance and cancer relapse because of their relative resistance to radiation, chemotherapy, and molecular targeted therapy (3). These residual cells after therapy have all the hallmarks of epithelial to mesenchymal transition (EMT) with increased metastasis capacity (3). Also, BCSCs are capable of proliferating in low adherence cell culture conditions in the presence of growth factors like epidermal growth factor and basic fibroblast growth factor to generate floating spheroids called tumorspheres or mammospheres (3). The number of spheroids represents the number of BCSCs, while their size depicts their proliferative capacity (3) (**Figure 2**).

**Figure 3:**
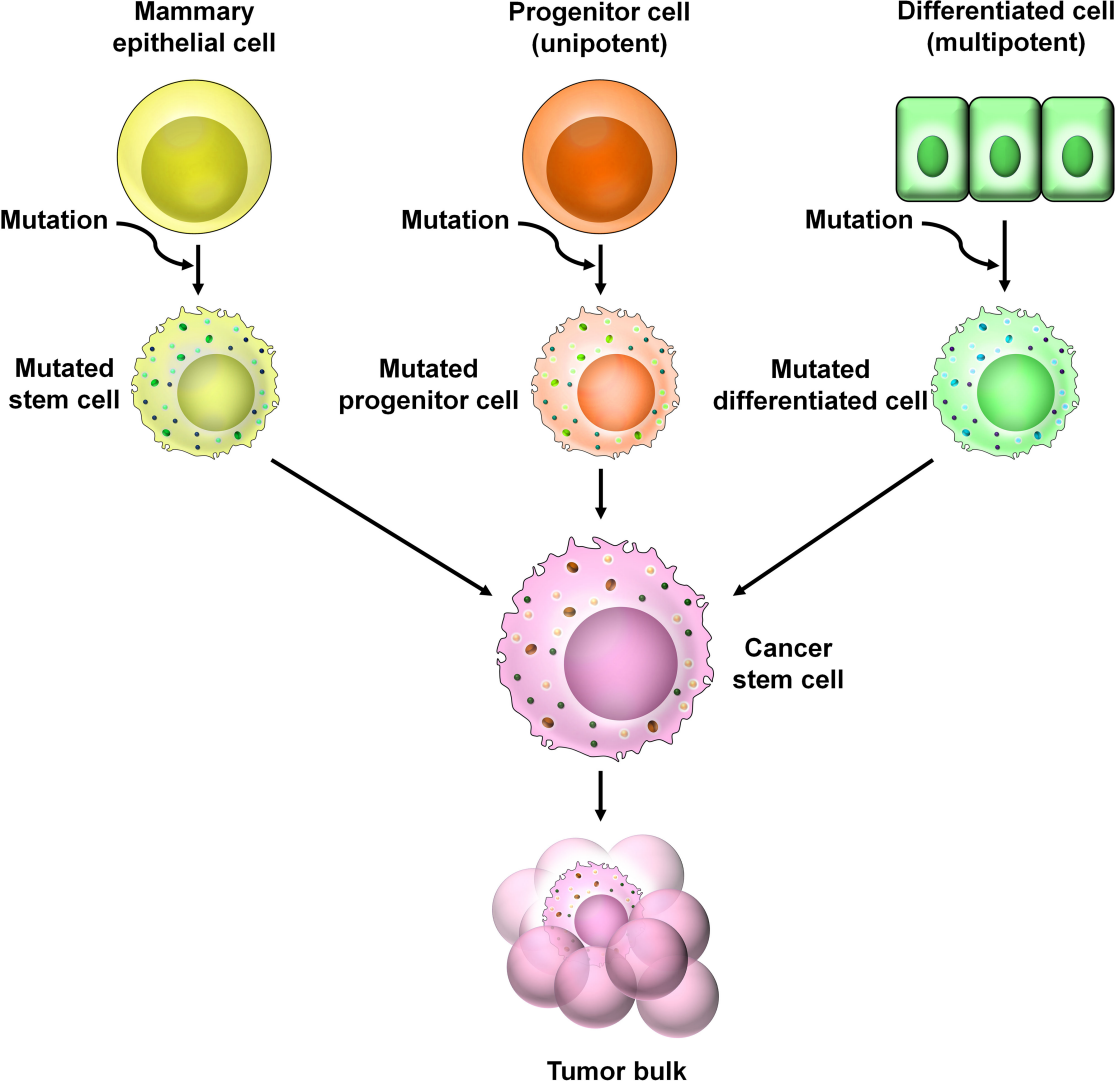
The origin of BCSCs. Numerous theories are prevalent regarding the origin of BCSCs. According to one theory, genetic and epigenetic alternations of non-stem cells within the TME cause the dedifferentiation of these cells into CSCs. A second theory suggests the presence of unipotent progenitor cells which accumulate mutations over time to give rise to CSCs. A third theory predicts that CSCs arise from multipotent mammary stem cells that have undergone mutational changes.

There are several theories regarding the origin of BCSCs (**Figure 3**). One theory supports that BCSCs arise from the dedifferentiation of non-stem cells (mammary epithelial cells) (4). Genetic and epigenetic alterations as well as changes within the tumor microenvironment (TME) contribute to the dedifferentiation of non-stem cells to the BCSC phenotype (4). Another theory suggests the presence of multipotent mammary stem cells as well as unipotent luminal and basal progenitor cells within the mammary glands (5). Accumulation of mutation in these progenitor cells may give rise to BCSCs. Growing bodies of evidence suggest that BCSCs can arise from normal stem cells by accumulating mutations (6, 7).

**Figure 4:**
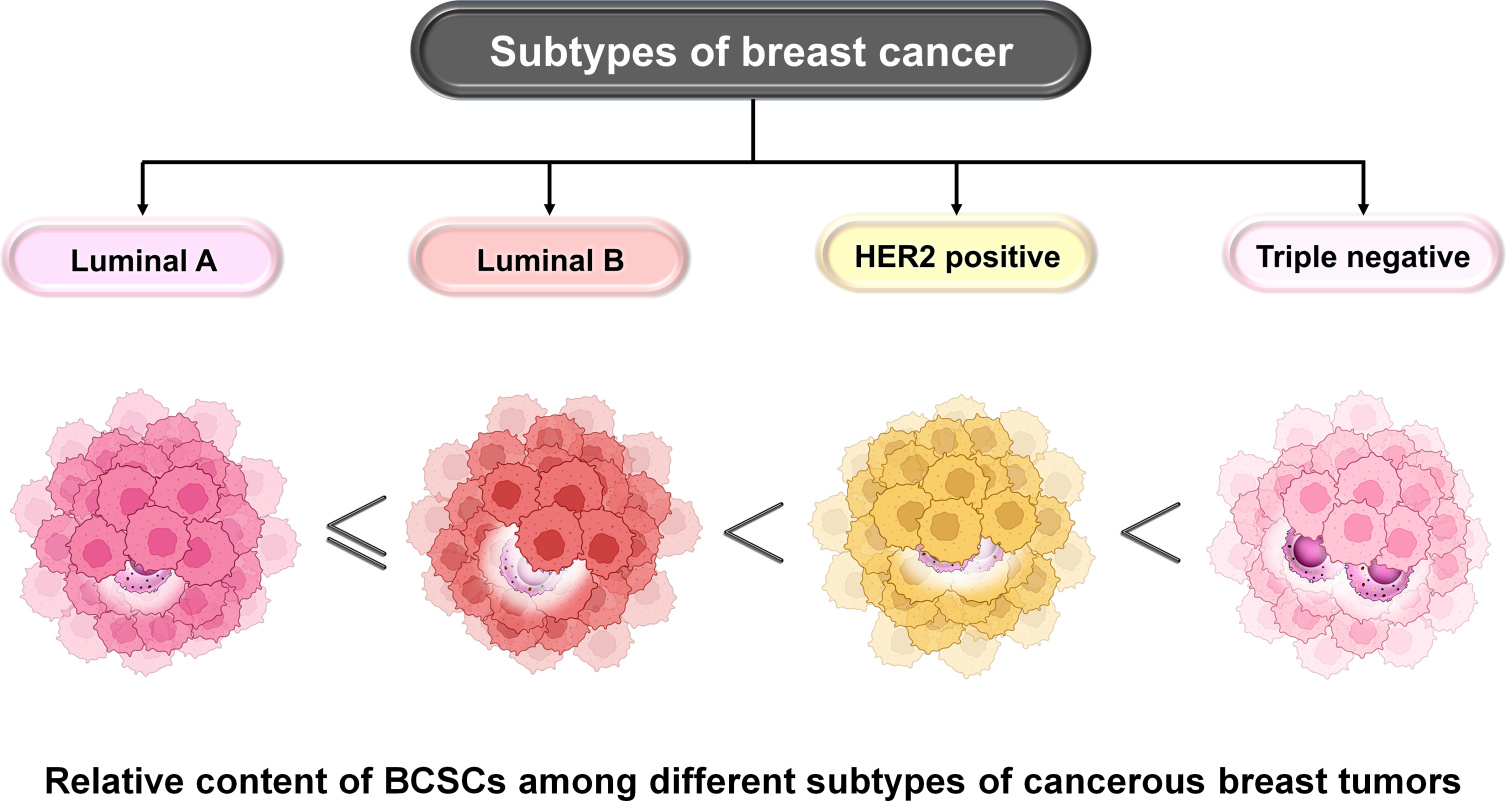
Relative content of BCSCs among different subtypes of BC. The proportion of BCSCs varies among different subtypes of BC and this correlates with their prognosis. Luminal A has the lowest proportion followed by luminal B, HER2^+^, and TNBC subtypes of BC. The landscape of developing TME involves infiltration, adaptation, and/or alteration as well as crosstalk-dependent cellular evolution involving cancer cells, immune cells, and the extracellular matrix (ECM), which altogether determines the fate of the tumor. A significant body of evidence suggests that a bidirectional crosstalk is involved in developing TME. On the one hand, immune cells of the TME modulate stemness in BC cells, and on the other hand, cancer cells escape immune surveillance by exercising their effects on cells like tumor-associated macrophages (TAMs), dendritic cells (DCs), myeloid-derived suppressor cells (MDSCs), T regulatory (Treg) cells, natural killer (NK) cells, and tumor-infiltrating lymphocytes (TILs). The co-evolution of the TME and BCSCs determines the fate of BC.

BCSCs are characterized based on the expression of several markers derived from breast cancer cell lines, transgenic mouse models, and patient-derived tumors. Among these, the most commonly used markers are CD44^+^/CD24^−/low^ and alcohol dehydrogenase 1 (ALDH1^+^). ALDH belongs to the family of NAD(P)^+^-dependent enzymes which are involved in the detoxification of a wide variety of aldehydes to their corresponding carboxylic acids. It mainly functions in converting vitamin A (retinol) to retinoic acid (8–10). It maintains the characteristics of cancer stem cells including drug resistance, thereby contributing to disease relapse (11). CD44^+^/CD24^−/low^ and ALDH1^+^ represent two distinct subpopulations of BCSCs that are different from one another (9). These two states are highly dynamic and interchangeable (9). Their number varies among different subtypes of BC. Among all subtypes, luminal-A BC has the lowest proportion of BCSCs which contributes to its best prognosis (9). Luminal B, however, has higher proportions than luminal A but lesser than TNBC or HER2^+^ breast cancers (**Figure 4**). The HER2^+^ BC is characterized by the presence of ALDH1^+^ epithelial BCSCs leading to its poor prognosis. TNBC is the most heterogeneous subtype and is characterized by the presence of the highest population of BCSCs (12–14). Claudin-low TNBC has a high proportion of mesenchymal BCSCs (CD44^+^/CD24^−/low^), whereas the basal-like TNBC has higher proportions of ALDH1^+^ epithelial BCSCs and also certain amounts of mesenchymal BCSCs (12).

In this review, we have tried to demonstrate the underlying mechanism of the crosstalk between BCSC with various immune cells of relevance, the major determinant factor of tumor fate. Focus has also been given to their impact on the plasticity of certain immune cells and the stemness of BC cells to keep immune surveillance in check so that tumor proliferation and progression can be encouraged without immune hassle. Finally, some BCSC-based therapeutic strategies have been discussed that might help in regulating BC severity.

## 2 Plasticity and heterogeneity of BCSCs

Cancer stem cells (CSCs) exhibit the capacity of self-renewal, initiating and promoting primary tumor growth, and can drive metastases at distal sites. In 2003, BCSCs were first isolated using cell surface markers for CD44^+^/CD24^−/low^. There exist two distinct developmental states for BCSCs, and due to cellular plasticity, these two states are reversibly interchangeable (9). One BCSC state is mesenchymal-like (CD44^+^/CD24^−^) with low proliferative activity and located mainly at the tumor-invasive edge (9, 15, 16). The other BCSC population is epithelial-like (ALDH1 expressing) which is highly proliferative and mainly found at the center of the tumor (9, 15, 16). Cytokine signaling such as inhibitor of DNA binding 1 (Id1) reversibly regulates the transition between two BCSC states (9). BCSCs which are CD44^+^/CD24^−^ and ALDH^+^ have robust tumor-initiating capacity and are involved with the incidence of chemoresistance and breast cancer relapse. When small numbers of isolated BCSCs were injected into immune-compromised mice, they exhibited a high capacity to develop tumors compared to when the system was injected only with a high number of cancer cells (17). Tumors generated by BCSCs maintained the cell type heterogeneity of the primary tumor.

## 3 BCSCs and the tumor microenvironment

Research studies of the last two decades have replaced the traditional view of breast cancer as a homogeneous system of rapidly proliferating neoplastic cells and uncovered the true face of the disease and established that BC is composed of cancer cells along with a variety of immune and other host cells that altogether create the breast TME. The crosstalk between all these cellular and structural components in the BC TME is cardinal for the growth and progression of BC. Here, we are specifically focusing on the interactions between BCSCs along with various immune cells in order to understand the consequences of such interactions and their role in the determination of breast cancer fate.

### 3.1 Interaction between BCSC*s* and innate immune cells

#### 3.1.1 BCSC and NK cells

**Figure 5:**
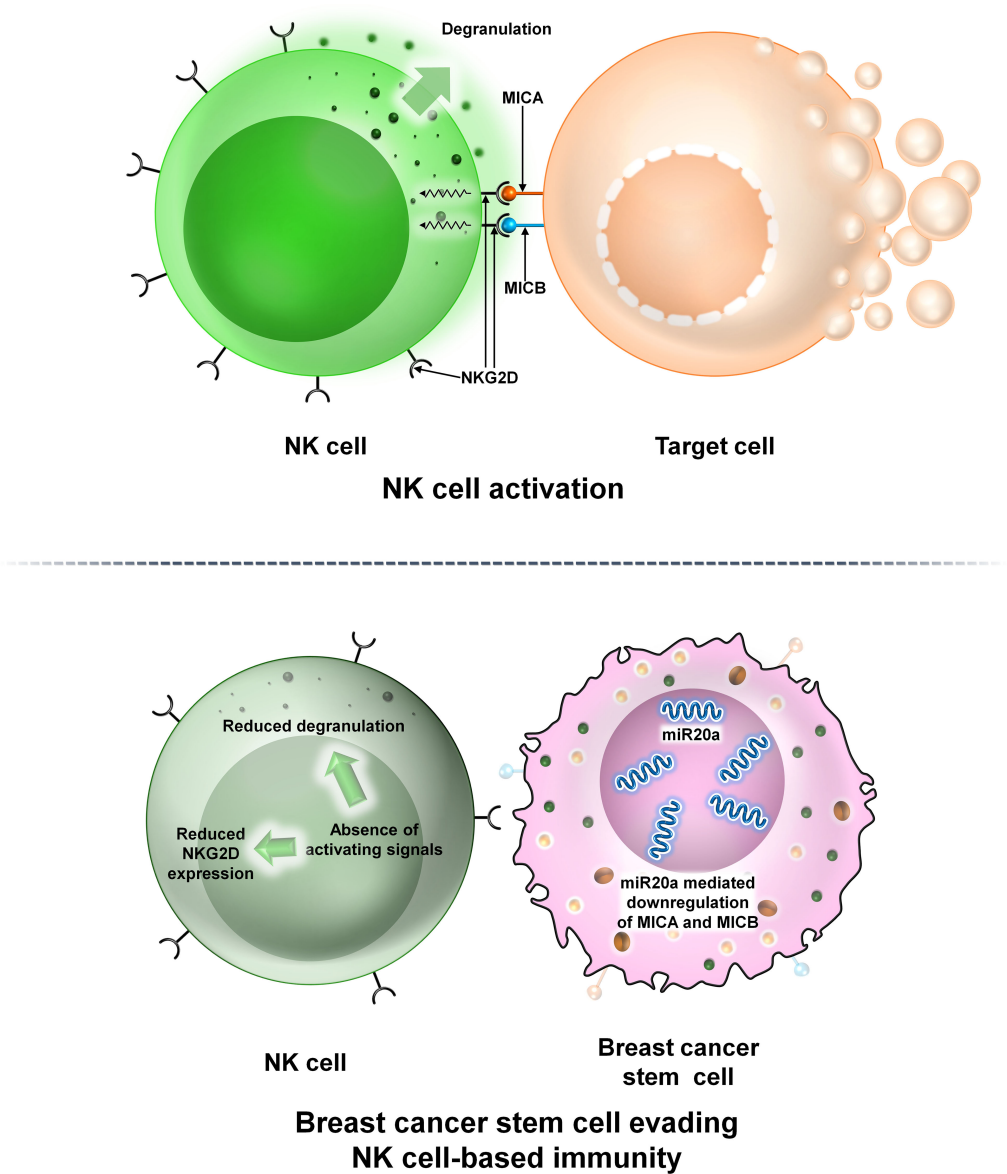
The interaction between NK cells and BCSCs. Representative image of the interaction between NK cells and BCSCs. Upper panel: The NK cell interacts with the target cell via NKG2D. NKG2D interacts with its two ligands MICA and MICB on the surface of the target cell thereby mediating NK cell cytotoxicity. Lower panel: Interaction of NK cells with BCSCs causes reduction of MICA and MICB on the surface of BCSCs via miR20a. This prevents the functioning of NKG2D thereby preventing degranulation of NK cells and allowing BCSCs to escape NK cell-mediated cytotoxicity.

NK cells are large granular lymphocytes constituting 5%–10% of circulating lymphocytes in human peripheral blood and take part in both innate and adaptive immune features (18, 19). NK cells interact with tumor cells or other components to modulate tumor growth within the TME (20). In BCs, ALDH^+^ BCSCs are resistant to NK cell cytotoxicity. BCSCs downregulate the expression of major histocompatibility complex I (MHC-I) chain-related proteins A and B (MICA and MICB) (21). MICA and MICB are the two ligands required for the functioning of the NK-activating receptor NKG2D. This ultimately reduces NK cell cytotoxicity, allowing tumor cells to escape immune surveillance and promotes metastasis (21). A recent study reveals that overexpression of miR20a reduces the levels of MICA and MICB within BCSCs, facilitating BCSCs to escape NK cell-mediated killing (21) (**Figure 5**).

#### 3.1.2 BCSCs and dendritic cells

**Figure 6:**
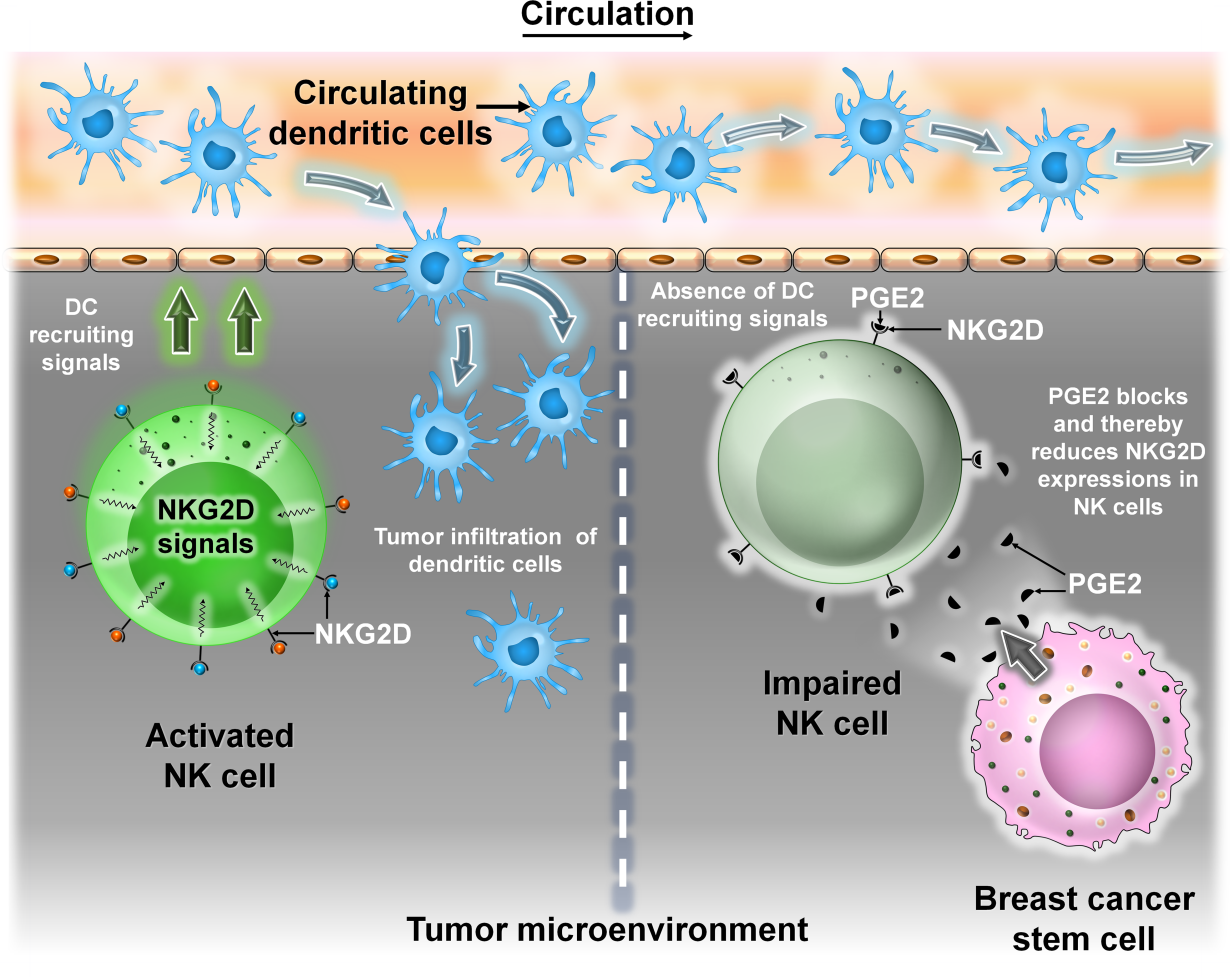
The interaction between DCs and BCSCs. Representative image of the interaction between DCs and BCSCs. Left panel: Recruitment of circulating DC into the TME is mediated by NKG2D signals from NK cells. Right panel: BCSCs block the activity of NKG2D by secreting prostaglandin E2 (PGE2), thereby preventing NK cell-mediated recruitment of DC into the TME.

DCs, first discovered by Steinman and Cohn (22), are professional antigen-presenting cells (APCs) and express high levels of both MHC class I and MHC class II molecules (23). DCs can be found in both “immature” and “mature” states. Mature DCs (M-DCs) can be distinguished from immature ones (i-DCs) by their ability to activate naive T cells in secondary lymphoid organs (24). The TME is enriched with different subsets of DCs like plasmacytoid DC, conventional type 1 and conventional type 2 DCs, and monocyte-derived DCs (25). The maturation state and density of these cells correlate with disease prognosis. In the TME, DCs interact with the other immune cells or stromal cells. This interaction can either induce or inhibit DC functioning (26). In a recent study, it was shown that in a mouse breast cancer model, tumor cells impair the recruitment of DCs into the TME by secreting prostaglandin E2 (PGE2). PGE2 downregulates the expression of the NK cell activating receptor NKG2D, thus impairing NK cell-mediated DC recruitment into the TME (27, 28). Though not much has been done regarding the interaction of DCs with BCSC, studies have shown that dendritic cells secrete milk fat globule EGF-8 (MFG-E8), which promotes specific CSC chemoresistance in BC cells (**Figure 6**).

#### 3.1.3 BCSCs and macrophages

Macrophages arise from monocytes which migrate from circulation to tissues where they differentiate into tissue-specific macrophage (29). Macrophages can be broadly divided into two categories: M1 macrophage (secretes proinflammatory cytokines like IL-12, IL-23, and TNF-α) and M2 macrophage [produce anti-inflammatory cytokines like IL-10 and transforming growth factor β (TGF-β)] (29–31). Tumor-associated macrophages (TAMs) play a dual role in the TME by secreting cytokines to promote tumor progression and by expressing immunosuppressive receptors to dampen immune surveillance. A crosstalk between CSCs and TAMs occurs in the TME which promotes survival and maintenance of CSCs.

**Figure 7:**
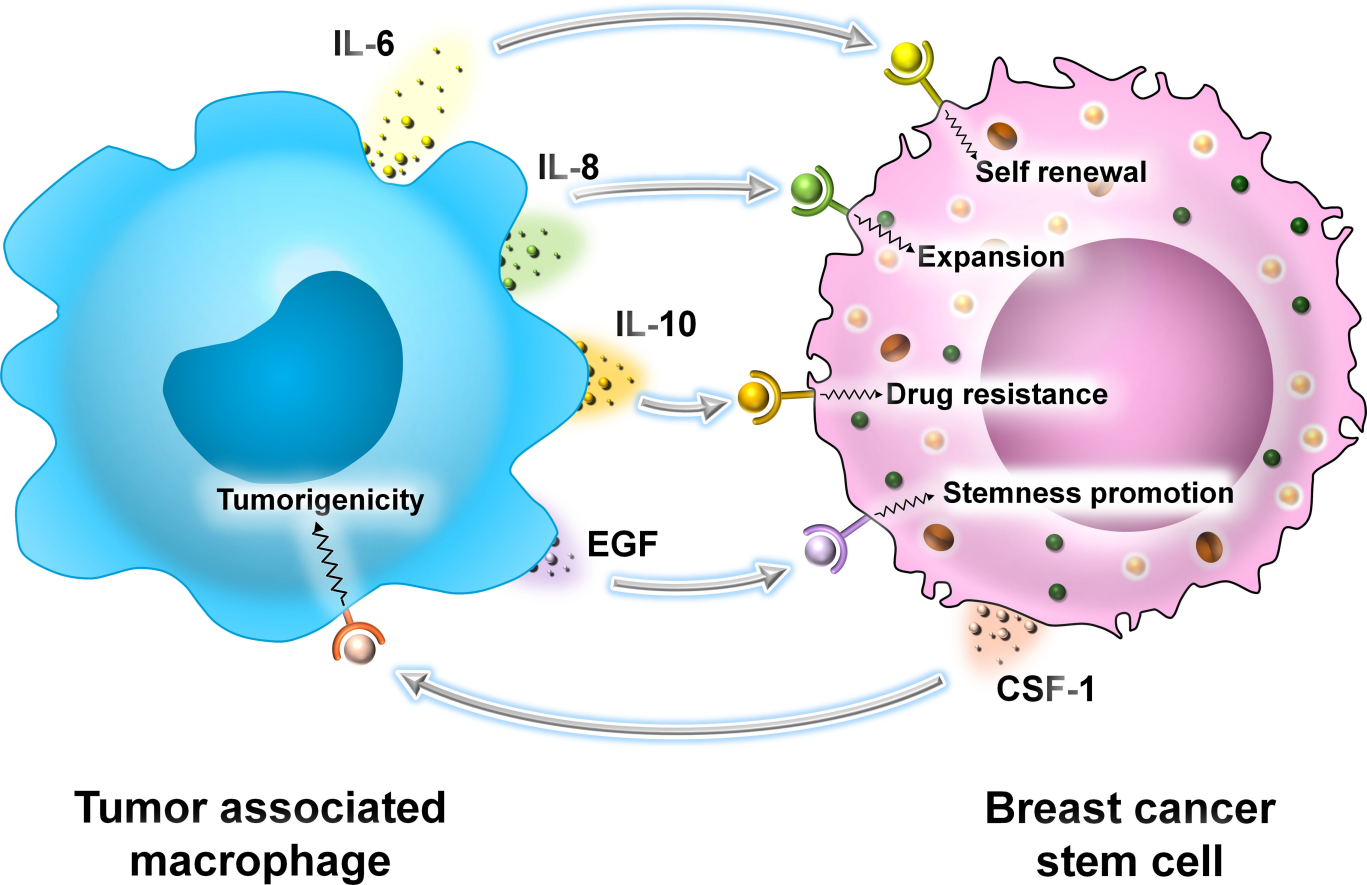
The interaction between TAMs and BCSCs. Representative image of the interaction between TAMs and BCSCs. TAMs secrete cytokines like IL-6, IL-8, and IL-10 and growth factor EGF, promoting self-renewal, expansion, drug resistance, and overall stemness in BCSCs, respectively. BCSCs, on the other hand, secrete CSF-1 that promotes the tumorigenicity of TAMs.

In BC, TAMs produce proinflammatory cytokines like IL-6 and activate the STAT3 signaling cascade to promote self-renewal of BCSCs (32–35). Secondly, IL-8 produced by TAMs promotes the expansion of BCSCs and prevents their programmed cell death (33, 35). TAMs secrete IL-10 which contributes to resistance of BCSCs to chemotherapeutic drugs by activation of STAT3 and its downstream signaling components (36). Tumor cells with metastatic capacity are known as circulating tumor cells (CTCs) (33). Lin and colleagues in 2001 demonstrated that TAMs play an important role in metastasis. A small population of CTCs with CSC properties interacts with TAMs. TAMs induce an EMT in these cells by the activation of the STAT3 signaling cascade (33–37). Thus, the interaction between CSCs and TAMs is beneficial for promoting their survival within the TME. TAMs promote the stemness of CSCs, and in turn, CSCs enhance the tumorigenicity of TAMs by secreting CSF-1. TAMs secrete cytokines like IL-6, IL-8, and EGF to activate downstream signaling cascades like JAK/STAT, Akt, and NF-κβ, thereby promoting the stemness of CSCs (33) (**Figure 7**).

#### 3.1.4 BCSCs and MDSCs

**Figure 8:**
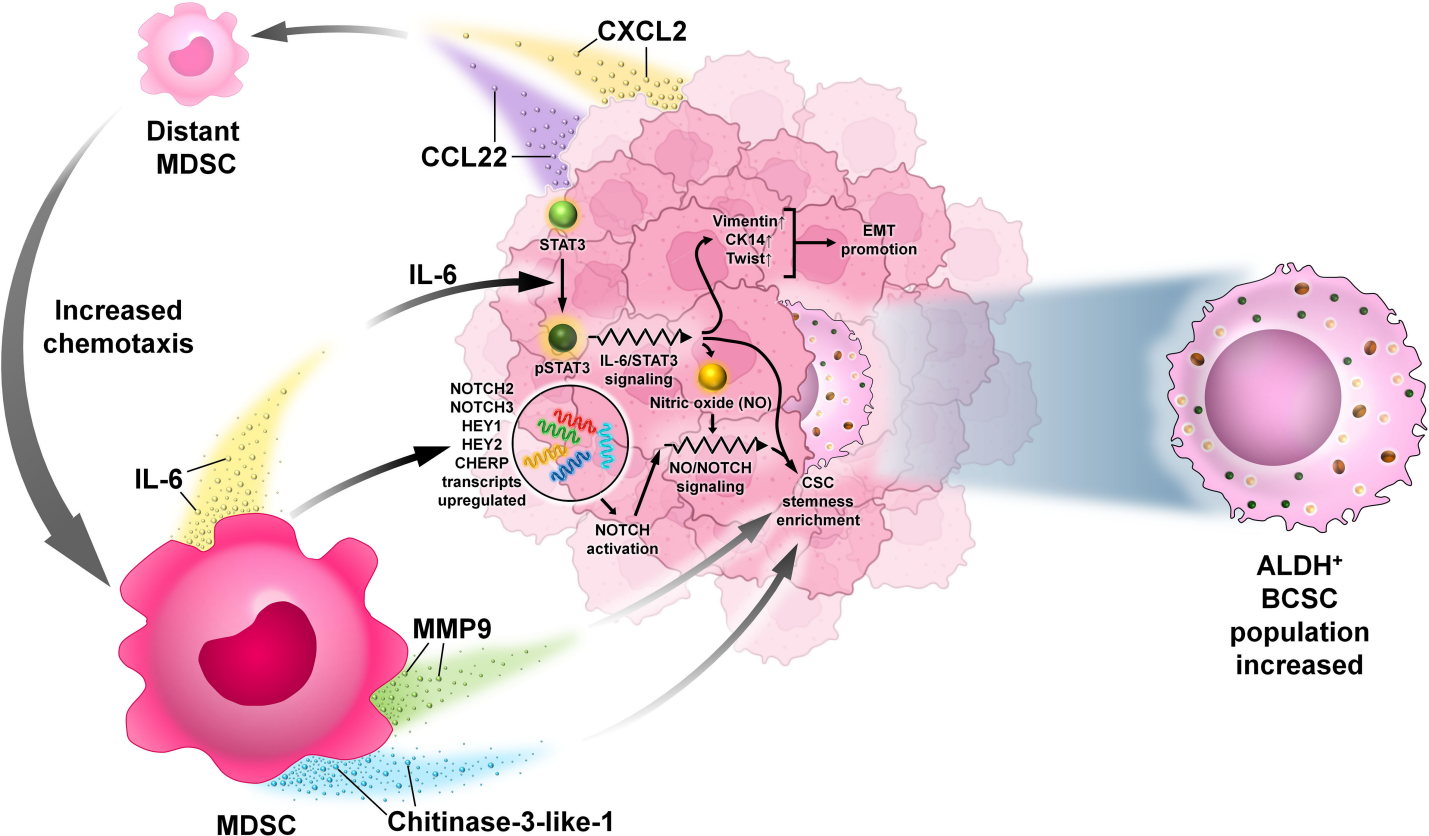
The interaction between MDSCs and BCSCs. Representative image of the interaction between MDSCs and BCSCs. MDSCs are recruited to the tumor site by ΔNp63-dependent activation of chemokines CXCL2 and CCL22. They secrete pro-metastatic factors such as MMP9 and chitinase 3-like 1 to induce BCSC enrichment. MDSCs cause IL-6-dependent phosphorylation of STAT3 that promotes NOTCH signaling (NOTCH2, NOTCH3, HEY1, HEY2, and CHERP transcripts and the intracellular domain of NOTCH) expression through nitric oxide (NO), leading to persistent STAT3 activation that results in the induction of EMT with high expression genes like Vimentin, CK14, and TWIST.

MDSCs are immunosuppressive cells and are best characterized by their ability to suppress T-cell-mediated immune response (38). These cells were first reported almost 30 years ago and are important in regulating immune response during various pathological conditions like autoimmunity, cancer, and infections (38, 39). They can be classified into two broad categories: polymorphonuclear MDSCs (PMN-MDSCS) and monocytic MDSCs (mMDSCs) (38, 39). MDSCs have been found to play a significant role in supporting BCSCs and in escaping the immune response. Ouzounova et al. reported that among the heterogeneous population of MDSCs, the monocytic and granulocytic have definite molecular properties. mMDSCs activate the STAT1 and STAT3 signaling pathways in tumors that result in the induction of the EMT/CSC phenotype characterized by the high expression of EMT-related genes like Vimentin, CK14, and TWIST. EMT is the phenomenon by which epithelial cells lose their characteristic features like cell adhesion and apicobasal polarity and gain migratory and invasive capacities like mesenchymal cells (40). This facilitates their voyaging through circulation to a new location, where they undergo the reverse mesenchymal to epithelial transition (MET) to generate secondary tumors (40, 41). This voyaging of tumor cells through circulation is termed as “metastasis” (40, 41). Metastatic human breast cancer samples compared with non-metastatic tumors also showed more infiltration of CD14-positive (human mMDSC marker) cells. MDSCs produce extrinsic signals for CSC renewal and raise tumor metastatic and tumorigenic potential. MDSCs influence CSC biology through three IL-6-dependent phosphorylation of STAT3 and nitric oxide (NO)-mediated NOTCH signaling pathways which maintain continuous and potent IL-6/STAT3 activation and impact cancer stemness. It was reported that MDSCs strongly induce STAT3 phosphorylation in breast cancer cell lines like MCF-7 and MDA-MB-231 co-cultured with MDSCs. NOTCH was activated in MCF-7 breast cancer cells by MDSCs by upregulation of NOTCH2, NOTCH3, HEY1, HEY2, and CHERP transcripts and of the intracellular domain of NOTCH (NICD) expression. MDSCs cause IL-6-dependent phosphorylation of STAT3 and trigger NOTCH signaling through NO, leading to persistent STAT3 activation (42, 43). MDSCs are associated with CSC content in the human BC microenvironment and correlate negatively with patient survival (42). MDSCs are reported to enhance the expression of various genes related to stemness to increase the human ALDH^+^ BCSC population by suppression of T-cell activation (**Figure 8**). A crosstalk between the STAT3 and NOTCH pathways in cancer cells has been reported (44). ΔNp63 was reported to enhance CSC activity by Kumar et al. as the number of generated tumorspheres decreased in ΔNp63-KD HCC1806 cells as compared with control (43, 45). MDSCs were recruited to the tumor site by direct ΔNp63-dependent activation of chemokines CXCL2 and CCL22 (43, 45). MDSCs secrete pro-metastatic factors such as matrix metalloproteinase 9 (MMP-9) and chitinase 3-like 1 to induce CSCs (43). However, it is unsettled whether MDSCs are related to CD44^+^/CD24^−^ BCSCs.

#### 3.1.5 BCSCs and neutrophils

Neutrophils are the most common type of white blood cells (WBCs) in circulation and play an important role in inflammatory response. They have a short life span of approximately 8 h only and make up to 40%–60% of the WBCs and are an essential component of the innate immune response. In cancer, neutrophils exhibit both pro- and antitumor responses upon infiltrating into the TME. These infiltrating neutrophils are called as “tumor-infiltrating neutrophils” (TINs). Like TAMs, TINs undergo polarization into N1 (antitumor) or high-density neutrophils and N2 (protumor) or low-density neutrophil phenotypes depending on the arriving signals. N1 neutrophils have an immune-stimulatory effect (46–48). They secrete proinflammatory cytokines like IL-12 and TNF-α which recruit CD8^+^ T cells.

In contrast, N2 neutrophils have an immune-suppressive effect and promote tumor progression by secretion of MMPs, reactive oxygen species (ROS), reactive nitrogen species (RNS), etc. (46). Recent studies have revealed that TINs at the early stage of the disease exhibit the N1 phenotype and are present at the margin of the tumor. As the disease progresses, TINs infiltrate into the center of the tumor and are converted into the N2 phenotype. This switch between N1 and N2 states are brought about mainly by the two cytokines TGF-β and type-I interferons produced in the TME. The presence of TGF-β polarizes TINs to the N2 phenotype, whereas in the presence of class I interferons, neutrophils are skewed toward the N1 phenotype (46).

In BC, the presence and number of TINs are used to determine the intensity of the disease. The presence of TINs contributes to poor disease prognosis. Patients with advanced stage of various cancers exhibit an increase in the ratio of absolute neutrophil to absolute lymphocyte in peripheral blood (NLR) (46, 49).

**Figure 9:**
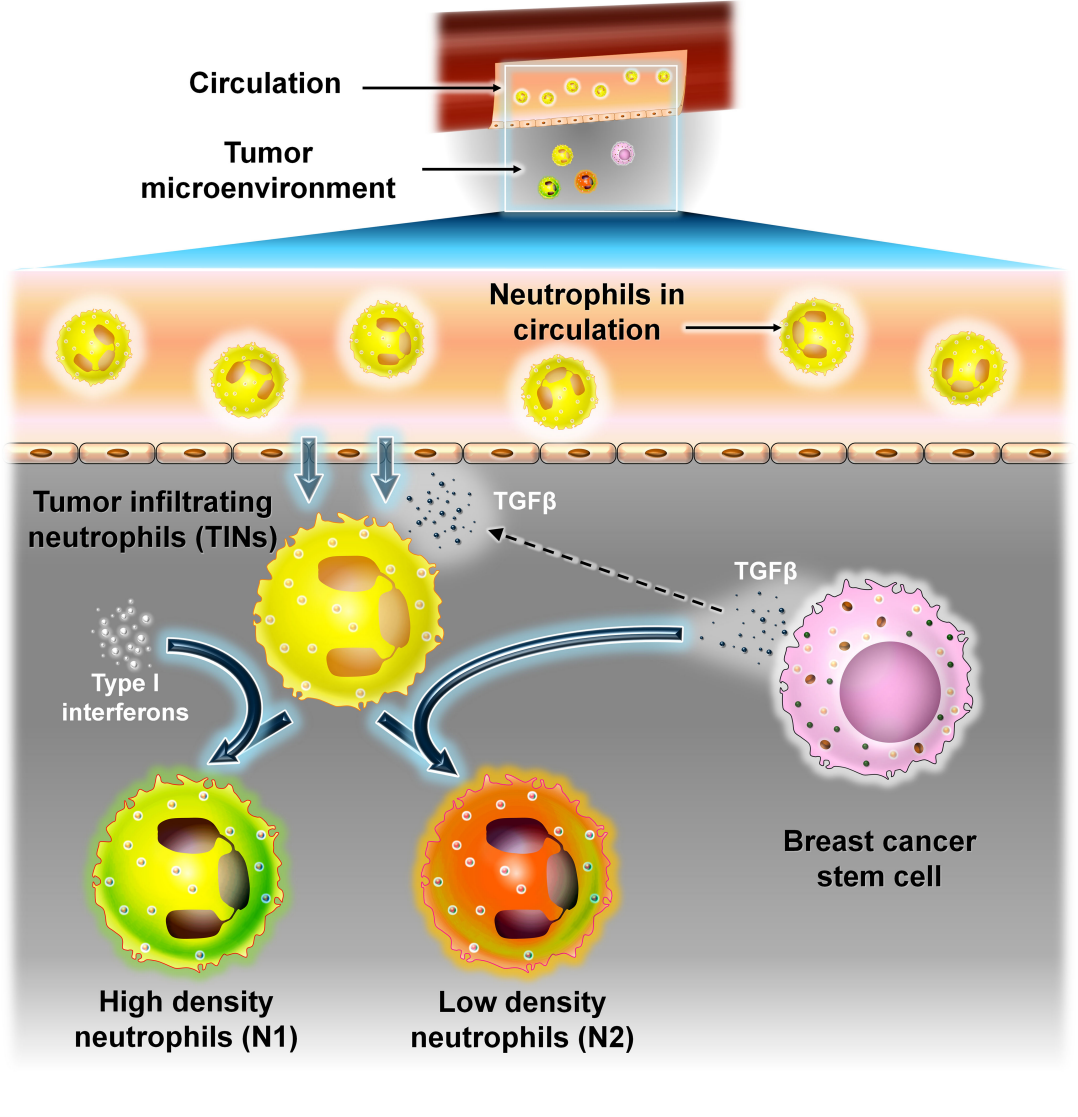
The interaction between neutrophils and BCSCs. Representative image of the interaction between neutrophils and BCSCs. Recruitment of neutrophils into the TME from circulation is brought about by TGF-β secreted from BCSCs. TINs can polarize into N1 (antitumor) or high-density neutrophils or N2 (protumor) or low-density neutrophils depending on the signal. Type 1 interferons convert TINs to the N1 type, whereas TGF-β from BCSCs polarizes them to the N2 type.

The presence of TINs in BC is limited and found in the highest proportion in TNBCs. This may be due to the presence of certain cytokines in TNBCs which directly or indirectly stimulate the production of neutrophils from bone marrow and their migration into the TME (48, 49). One such cytokine is TGF-β which is highly expressed in TNBC (46, 50). Oncostatin M produced by TANs is induced by BC cells which in turn promotes EMT and the BCSC phenotype. TANs secreted by the cytokine tissue inhibitor matrix metalloproteinase 1 (TIMP-1) also encourage EMT and metastasis (**Figure 9**).

#### 3.1.6 BCSCs and eosinophils

Eosinophils are a type of granulated WBCs produced in the bone marrow by hematopoiesis and comprise approximately 1%–5% of the total white blood cell population. Their cytoplasm is filled with numerous granules containing enzymes and proteins with various functions. The recruitment of eosinophils into the TME is brought about by a plethora of factors produced by both the tumor cells as well as by the other immune cells in the TME. The infiltration of eosinophils into the TME is brought about by eotaxins (eotaxin-1, eotaxin-2, and eotaxin-3) that activate the CCR3 receptor which is highly expressed on human eosinophils (47). TAMs and mast cells produce vascular endothelial growth factor (VEGF) that recruits eosinophils into the TME (47, 51).

Mast cells are immune cells of myeloid lineage and are present in all vascularized tissues (52). They are important for maintaining vascular hemostasis, allergy, anaphylaxis, cardiovascular diseases, and cancer (52). Their cytoplasm contains numerous granules which are filled with various inflammatory cytokines, histamine, heparin, etc. They are important in maintaining normal physiological conditions as well as during pathological conditions (52). IL-4 secreted by T helper 2 (Th-2) cells promotes indirect recruitment of eosinophils by inducing the local production of eotaxin-1 (47). Th-2 cells as their name suggests provide helper function to other immune cells like dendritic cells, macrophages, and B cells (53). They are subsets of CD4^+^ Th cells. They are mainly involved in type 2 immune response which is important in the eradication of parasitic and bacterial infections (53).

In cancer, eosinophils also have both protumorigenic and antitumorigenic roles. Tumor-infiltrating eosinophils produce cytokines that attract CD8^+^ T cells and induce M1 macrophage polarization which ultimately promotes inflammation and phagocytosis. Eosinophils can act in a protumorigenic manner. They produce MMP-9 that promotes metastasis, polarize macrophage to the M2 phenotype, and promote angiogenesis by secretion of VEGF, platelet-derived growth factor (PDGF), and fibroblast growth factor (FGF).

VEGF is the key mediator of angiogenesis in cancer. The formation of new vasculature in and around the tumor supplies oxygen and nutrients to the rapidly dividing cancer cells. However, the blood vessels so formed under the influence of VEGF are irregular in shape with dead ends, are leaky, and are not organized into venules, arterioles, and capillaries. These leaky vasculatures lead to suboptimal blood flow, thereby producing a hypoxic environment that further stimulates VEGF production (54).

PDGF as the name implies was first purified from platelets and promoted the proliferation of different types of cells like fibroblasts, smooth muscle cells, and glial cells. In cancer, autocrine PDGF signaling promotes proliferation, survival, metastasis, and angiogenesis. Also, paracrine PDGF signaling recruits stromal cells into the TME, which supports tumor growth and survival (55).

FGF is involved in diverse biological processes in both normal cells and tumor cells. They promote motility and invasiveness in various types of cancer. FGF-1 (acidic FGF) and FGF-2 (basic FGF) along with their receptors control the growth of malignant tumor cells in both autocrine and paracrine manner. FGF acts synergistically with VEGF to promote tumor angiogenesis (56, 57).

**Figure 10:**
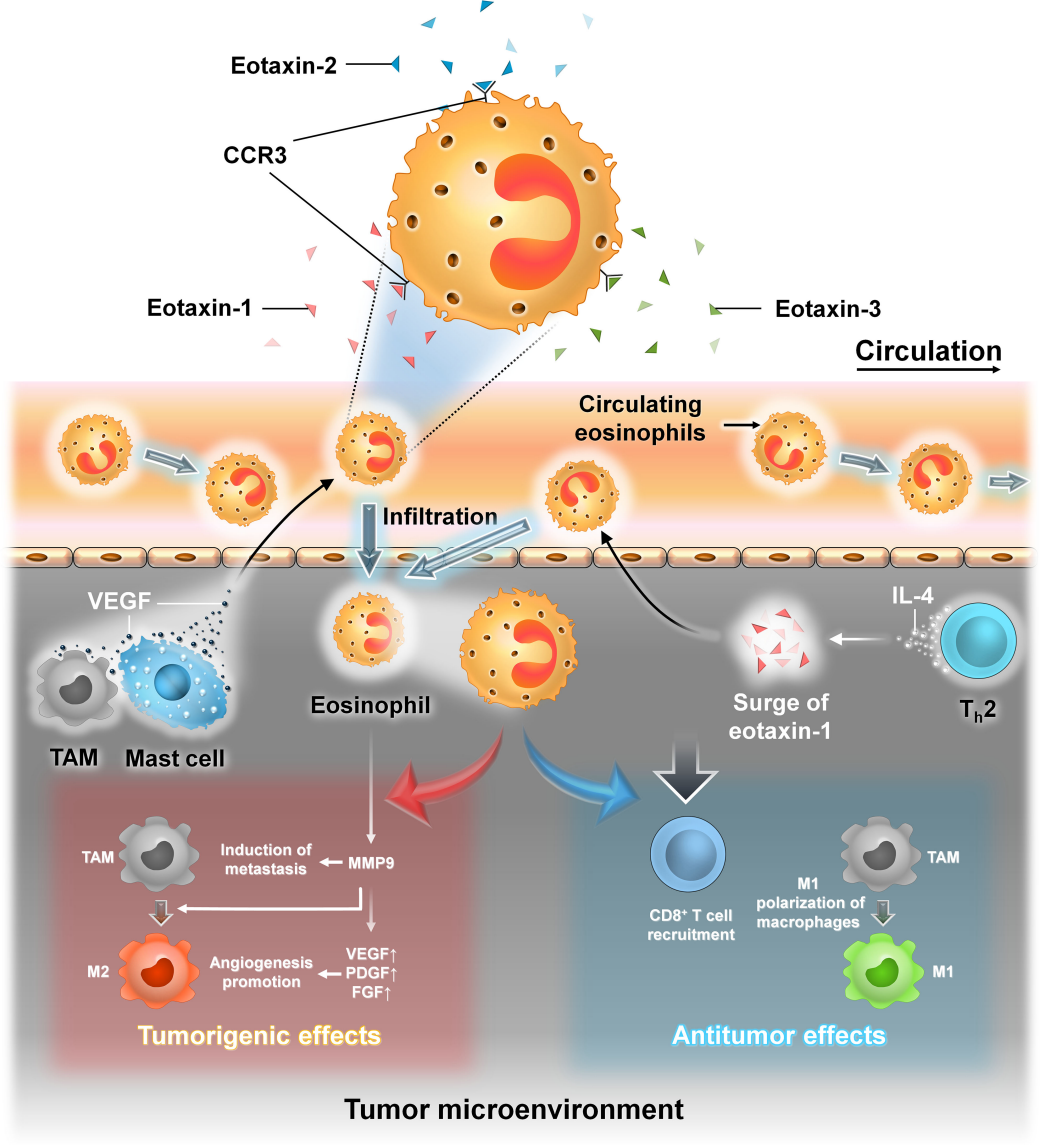
The interaction between eosinophil cells and BCSCs. Representative image of the interaction between eosinophils and BCSCs. Left panel: Recruitment of eosinophils into the TME from circulation is brought about by eotaxin-1, eotaxin-2, and eotaxin-3 that activate CCR3 on the surface of eosinophils. Also, VEGF secreted by TAM and mast cells recruits eosinophils into the TME. In the TME, eosinophils produce MMP-9 that promotes metastasis, polarizes TAM to the M2 phenotype, and also favors angiogenesis by secretion of VEGF, PDGF, and FGF, thereby exerting protumorigenic effects. Right panel: Indirect recruitment of eosinophils into the TME is mediated by IL-4 from Th-2 cells by the local production of eotaxin-1. In the TME, they promote the recruitment of CD8^+^ T cells and induce M1 macrophage polarization, thereby exerting antitumor effects.

Eosinophils are significantly associated with better outcome in ER^+^ breast tumor patients. In a study involving a mice model, intranasal administration of IL-33 reduced the number of lung metastasis by recruitment of eosinophils to the tumor site (47, 51). IL-33 is an important cytokine that induces the production of proinflammatory cytokines and recruits NK cells, eosinophils, and CD8^+^ T cells into the TME (58). Moreover, IL-33 also promotes CCL5 production by eosinophils and CD8^+^ T cells that recruit NK cells to the tumor site (58). Another cytokine, IL-17E, promotes eosinophil expansion within the TME by promoting IL-5 production. The role of eosinophils in breast cancer, especially their interaction with BCSCs, needs further investigation. Eosinophils can be a potential biomarker; therefore, targeting these cells can be an effective anticancer therapy (**Figure 10**).

### 3.2 Interaction between BCSC*s* and adaptive immune cells

#### 3.2.1 BCSCs and T cells

**Figure 11:**
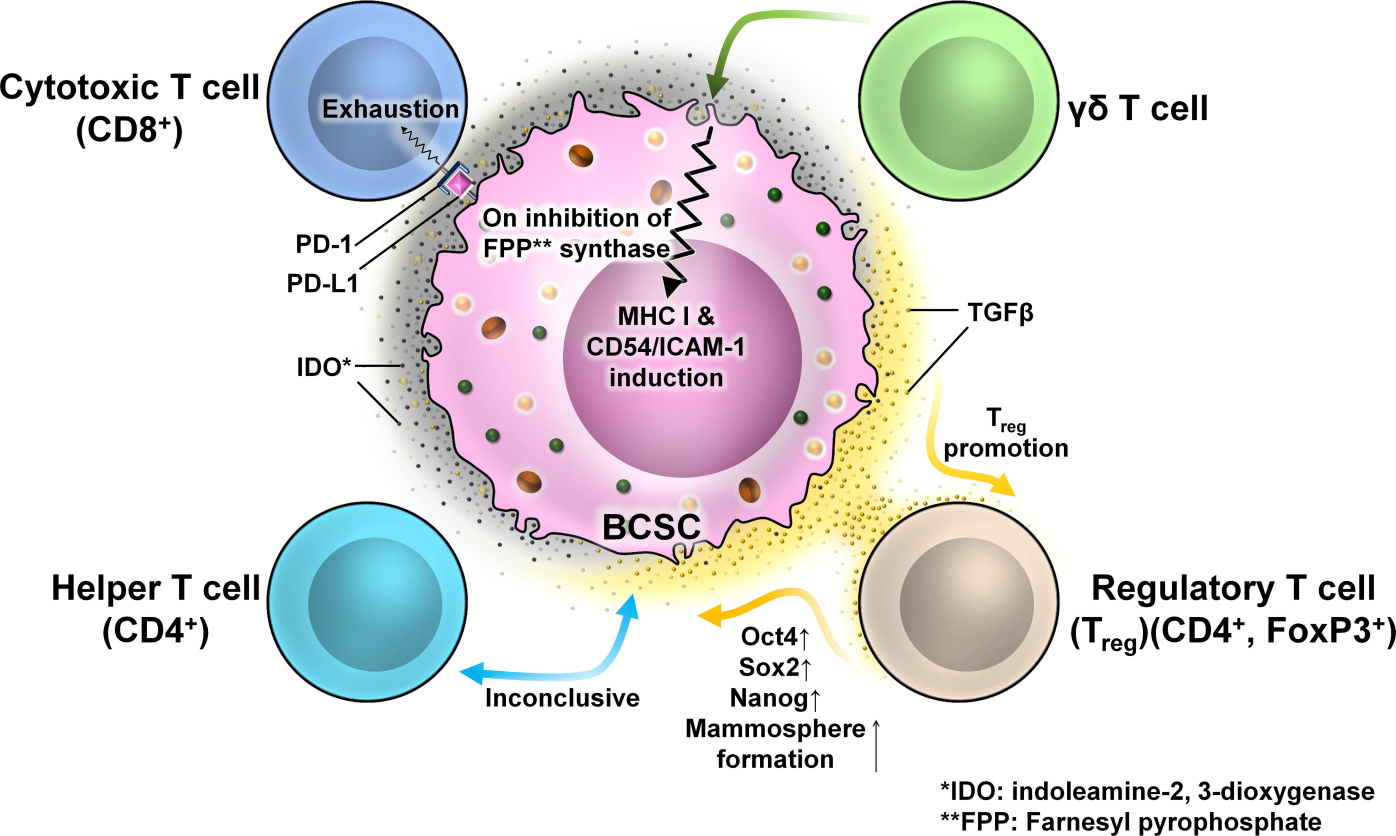
The interaction between different T cells and BCSCs. Representative image of the interaction between T cells and BCSCs. BCSCs express programmed death-ligand 1 (PD-L1) on their surface which interacts with programmed cell death protein 1 (PD-1) on the surface of CD8^+^ T cells to cause T-cell exhaustion. The interaction of BCSCs with CD4^+^ T cells is inconclusive. BCSCs have elevated levels of indoleamine-2,3-dioxygenase (IDO), which promotes the generation and activation of Tregs. Tregs regulate the stemness of BCSCs through the TGF-β signaling pathway that increased mammosphere formation with enhanced expression of OCT4, SOX2, and NANOG. BCSCs are resistant to γδ T-cell-mediated killing due to the presence of farnesyl pyrophosphate synthase. Inhibition of farnesyl pyrophosphate synthase results in MHC-I and CD54/ICAM-1 upregulation resulting in susceptibility to γδ T-cell- and CD8^+^ T-cell-mediated lysis.

T cells are components of the adaptive immune system and can acquire functional and effector properties depending on the immunological context. They are recruited to the tumor following activation within the lymph node upon receiving immunogenic stimulus. There are four T-cell subtypes relevant to breast tumor biology, namely, cytotoxic T cells, helper T cells, T regulatory cells, and γδ T cells. Infiltrations of CD8^+^ cytotoxic T cells are correlated with better tumor prognosis; however, with cancer advancement, this antitumor property gets blunt. In BC, high levels of TGF-β secretion by BCSCs cause dampening of the cytotoxic behavior of T cells causing immunological tolerance. BCSCs express programmed death-ligand 1 (PD-L1) having the capacity to bind programmed cell death protein 1 (PD-1) on effector T cells causing their exhaustion. Human TNBCs with high levels of PD-L1 expression also exhibit high stemness markers (e.g., OCT4 and NANOG) (**Figure 11**).

TILs are highly heterogeneous, which limits their responsiveness to immunotherapy against cancer (59). According to the international guidelines on TIL assessment, tumors with <10% TIL frequency are termed as “cold” tumors or poor immune-infiltrated tumors (59), whereas tumors with TIL frequency >10% are termed as “hot” tumors or inflamed tumors (59). The continuous antigenic stimulation and immunosuppressive environment within the TME cause CD8^+^ T cells to lose their proliferative and effector activities and enter an “exhausted” state (59). This helps the tumor cells to flourish unhindered.

Various immune checkpoint inhibitors like nivolumab (anti-PD-1) and atezolizumab (anti-PD-L1) have been used over the past decades to reboot these exhausted CD8^+^ T cells (59). Additionally, the presence of CD8^+^ T cells at the invasive tumor edges may facilitate an efficient immune response (60). However, the success rates are limiting which may be due to the variations within the target cells (59). Along with a substantial correlation between total mutational burden and clinical response, the expression of PD-L1 in tumor samples is correlated with the effectiveness of checkpoint blocking (61). Further studies on exhausted CD8^+^ T cells revealed that there are two subpopulations of these cells. Progenitor-exhausted CD8^+^ T cells (CD8^+^PD-1^+^TCF1^+^) respond to anti-PD-1 therapy, whereas terminally exhausted CD8^+^ T cells (CD8^+^PD-1^+^TCF1^−^) fail to respond to it (59).

A recent study with advanced breast carcinoma demonstrated that both progenitor and terminally exhausted CD8^+^ T cells are present in the TME with a positive correlation between terminally exhausted CD8^+^ T cells and BCSCs (59). Moreover, this correlation was more strongly elevated in cold advanced breast carcinoma than in inflamed tumors (59). Functional analysis of BCSCs revealed that in cold tumors, BCSCs had overexpression of OCT4, NANOG, ALDH1, and KLF4 with marginal elevation of SOX2 in comparison to hot tumors (59).

Furthermore, *in-vitro* studies using 3D spheroid culture revealed that the interaction of terminally exhausted CD8^+^ T cells with BCSCs generated highly aggressive, migratory, and invasive subtypes of BCSCs (59).

The most frequently utilized research techniques in breast cancer study include 2D monolayer cell cultures, patient-derived xenografts, and genetically modified murine models (62, 63). Compared with conventional research approaches, breast cancer organoids (BCOs) created from tumor tissues showed a number of advantages. In contrast to 2D culture systems, BCOs preserve the 3D structure of the tumor along with the tumor microenvironment and its biological components (62–64). They can be conserved as “biobanks” for future research due to their quicker generation time, increased passage efficiency, and overall cost effectiveness (62, 64). Because they offer a reliable and repeatable platform for carrying out high-precision tests, BCOs are frequently employed in clinical trials for drug screening and personalized therapy (62–64). Three types of 3D culture systems can be distinguished based on the material employed. Culture can be done in three different ways: on non-adherent plates, Matrigel-like materials, or scaffold-based systems (63).

In the co-culture setup of progenitor and terminally exhausted CD8^+^ T cells with BCSCs in 3D CSC enrichment setup for tumorsphere formation, it was disclosed that, in comparison to progenitor CD8^+^ T-cell-treated BCSCs, terminally exhausted CD8^+^ T-cell-treated BCSCs had a greater number of tumorspheres (59). These tumorspheres were aggressive, resistant to therapeutic agents, clonogenic, and invasive (59). The addition of PD-1–PD-L1 interaction inhibitor 1 (small molecule inhibitor (ab230369, ICI agent) reduced BCSC frequency with reduced tumorsphere formation in progenitor exhausted CD8^+^ T-cell-treated BCSCs but not in the terminally exhausted CD8^+^ T-cell-treated group (59).

The role of CD4^+^ T cells on BCSCs is inconclusive. One study with BC patients revealed that CD4^+^ T cells correlate positively to the presence of CD44^+^CD24^−^ BCSCs (65).

There are several disputes regarding the significance of Tregs in BC. One study revealed that hormone receptor-positive (HR^+^) breast tumor subtypes with high FOXP3 TILs correlate with poor prognosis and high grade (66). Several other studies revealed that FOXP3^+^ TILs are not an independent prognostic factor in HR^+^ BC. The effects of Tregs on CSCs are less understood. Tregs have a high and stable expression of TGF-β on their surface which is activated in CD44^+^ BCSCs. Tregs regulate the stemness of BCSCs through the TGF-β signaling pathway and increase mammosphere-forming ability. BCSCs also have elevated levels of indoleamine-2,3-dioxygenase (IDO) (67, 68), an immune-modulatory enzyme which suppresses the activation of T cells and promotes the generation and activation of Tregs (22). An experiment revealed that a Treg-conditioned medium increased the BCSC population within a murine BC cell line. These cells formed a greater number of spheres with enhanced expression of the genes associated with stemness like OCT4, SOX2, and NANOG (69, 70).

γδ T-cell populations are characterized by heterodimeric T-cell receptor (TCR) expression formed by γ and δ chains. They play a role in antitumor immune response. HMLER-derived CSC-like cells in the BC scenario are resistant to γδ T-cell-mediated killing, but inhibition of farnesyl pyrophosphate synthase results in MHC-I and CD54/ICAM-1 upregulation, which increases the susceptibility to γδ T-cell- and CD8^+^ T-cell-mediated lysis (71).

### 3.3 BCSCs and platelets

Platelets are tiny blood cells that develop from the bone marrow and play an important role in wound healing. They are anucleated cells and have a number of granules in their cytoplasm, namely, α-granules, dense granules, and lysosomes (72). The normal platelet count ranges from 150,000 to 450,000 platelets per microliter of blood. High platelet count is considered a risk factor associated with various tumors including BC. Inactivated platelets generally have a biconvex disc shape. Activated platelets have numerous filopodia processes covering their surface (72). Within the TME, platelets help cancer cells undergo EMT and metastasize to a new location where they can generate secondary tumors (73). In circulation, tumor cells train platelets to form tumor-educated platelets or TEPs that attach to tumor cells by integrins, fibrins, and P-selectins and enclose these tumor cells completely, thereby protecting the CTCs from attack by the immune cells of the body like NK cells in circulation and hence helping the tumor cells to escape the immune surveillance process (74, 75). However, recent studies on cancer stem cells have revealed that cancer stem cells are more efficient in activating platelets than the tumor cells themselves (76). In a study involving the mice breast cancer cell line 4T1, it was observed that spheroids obtained by growing 4T1 cells in serum-free media and low adherence condition were more efficient in activating platelets than the 4T1 cells themselves (76). These spheroids were more efficiently coated by platelets than by the 4T1 cells (76). The interaction between these platelets and cancer stem cells causes the release of TGF-β1 from the α-granules of platelets (76). TGF-β1 inhibits NK cell activity by downregulating the expression of NKG2D which belongs to the group of NK cell-activating receptors (76–78).

**Figure 12:**
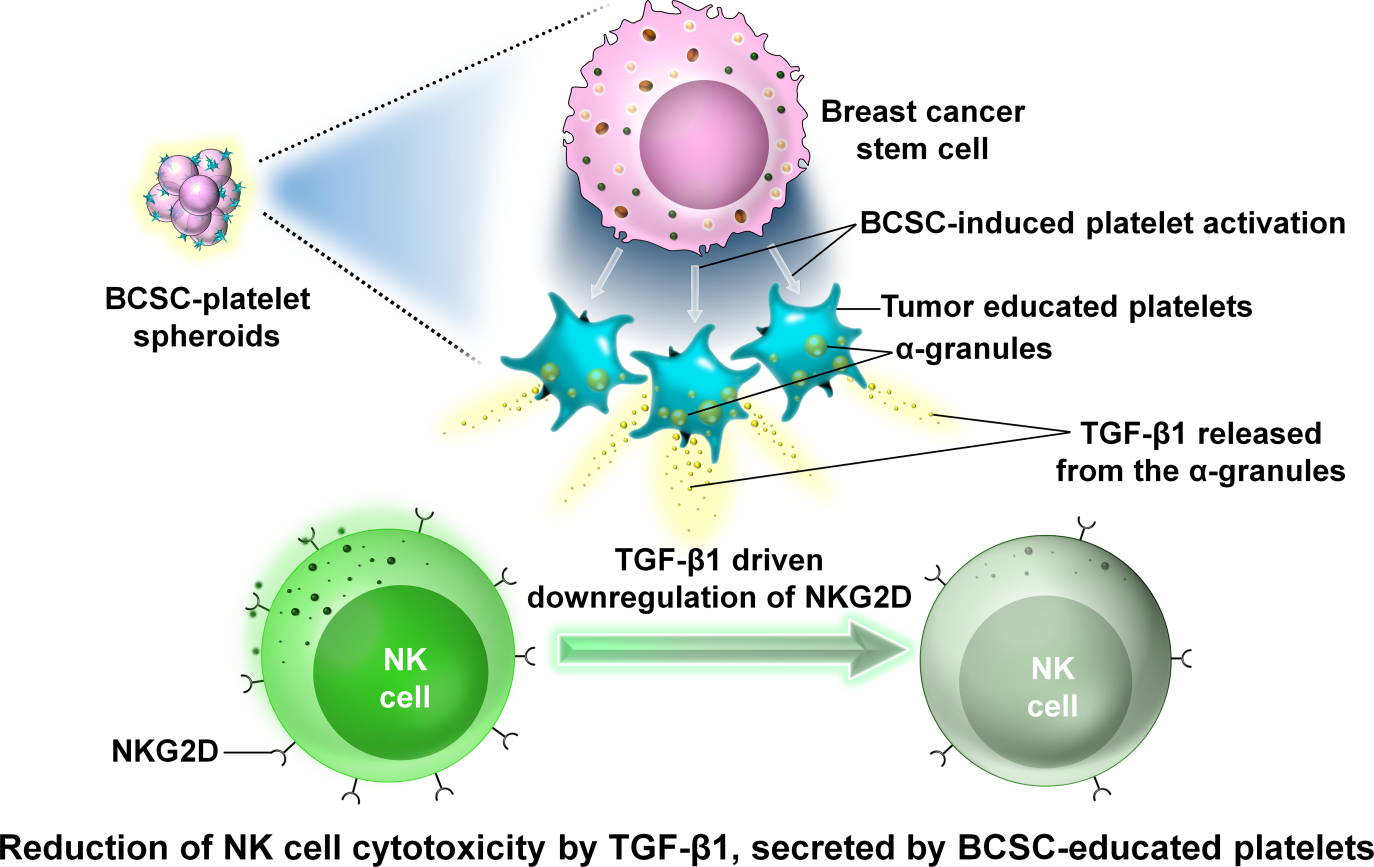
The interaction between platelets and BCSCs. Representative image of the interaction between platelets and BCSCs. The interaction between platelets and BCSCs causes the release of transforming growth factor β1 (TGF-β1) from the α-granules of platelets. TGF-β1 inhibits NK cell activity by downregulating the expression of NKG2D which prevents the antitumor activity of NK cells.

This downregulation prevents the antitumor activity of NK cells. Thus, platelets play an immense role in the survival of tumor cells in circulation (**Figure 12**).

### 3.4 BCSCs and CAFs

Cancer-associated fibroblasts (CAFs) are a key component of the TME and play critical roles in the co-evolutionary process of tumor stroma and, thus, tumorigenesis (79, 80). They produce collagens and fibronectins as well as ECM-degrading enzymes (79). CAFs enhance sphere formation, promote self-renewal and expansion of BCSCs, and induce the expression of the stemness markers SOX2, BMI-1, and CD44 (79). Also, CAF-treated CSCs enhanced tumorigenicity in *in-vivo* breast tumor models (79). Recent studies have identified a subpopulation of CAFs that secrete prostemness paracrine factors that promote the conversion of cancer cells to CSCs or they support self-renewal or stemness of existing CSCs in the tumor (81).

Various chemokines and cytokines secreted by CAFs regulate the stemness of BCSCs. SDF-1 secreted by CAFs interacts with CXCR4 on BCSCs and activates the WNT/β-catenin pathway to promote the generation of BCSCs with sustained tumorigenicity and metastatic activity in BC (79, 82). CCL2, another cytokine produced by CAFs, promotes self-renewal and expansion of BCSCs via the NOTCH1 pathway (79).

TGF-β produced by CAFs can regulate stemness directly by promoting EMT of BCSCs through the TGF-β/Smad axis. Indirectly, it induces the production of SDF-1 by CAFs, thereby regulating stemness via the SD1/CXCR4 pathway (79, 81, 82).

Therapeutic targeting of CAFs is essential since they have become one of the main factors regulating CSCs. Breast cancer treatments that directly deplete fibroblast activation protein (FAP)-positive CAFs using FAP-PE38 toxin-conjugated antibodies have shown good outcomes (79). Preclinical experiments that targeted the CAF–CSC signaling pathway also produced notable outcomes (79). BCSC stemness was decreased by blocking IL-6/IL-6R/STAT3 signaling with the STAT3 inhibitor Stattic (79). In preclinical studies, the additional use of NOTCH, TGF, and Hedgehog inhibitors has demonstrated potential outcomes in lowering BCSC stemness (79, 81).

## 4 CSCs as a key player in immune evasion of breast cancer

The interaction between cancer cells and immune cells is highly complex and can be both beneficial and detrimental for tumor cells. The final outcome of this interaction depends on various environmental stimuli within the TME. “Escape of immune surveillance” is one of the major hallmarks of tumor cells, and they use various techniques like secretion of immune-suppressive molecules like IL-6 and TNF-α, while others edit the host immune system by targeting the regulatory T cells. Defective antigen presentation by targeting the MHC-I pathway is another strategy used by tumor cells to escape the host immune system (78). CSCs also are endowed with properties that help tumors in immune evasion (83). CSCs may secrete immunosuppressive factors and also recruit non-cancerous immune-suppressive cells (83). CSCs may downregulate MHC molecules and tumor-associated antigens, thereby inhibiting/escaping from the immune response of T cells (83).

The two types of BCSCs—ALDH1^+^ and CD44^+^/CD24^−^—have different mechanisms to escape the immune response. ALDH1^+^ CSCs downregulate the TAP gene and CD80, thereby targeting the antigen presentation pathway (84), while the CD44^+^/CD24^−^ group upregulates the expression of CXCR4 which is involved in EMT and the induction of the CSC phenotype in BC (83, 85, 86). BCSCs are also capable of inhibiting various cytolytic granules like perforin and granzyme B. Several studies support that CSCs from estrogen receptor-α-positive cell line inhibit granzyme B by upregulating the expression of protease inhibitor 9 which targets granzyme B (87, 88). CD44^+^/CD24^−^ BCSCs express higher levels of TGF-β (a potent inducer of EMT) than the non-CSC population (84, 89, 90). Moreover, Sca-1 is found to be involved in TGF-β signaling (88, 90). In a human BC model, Sca-1 homologs Ly6K and Ly6E play important roles in TGF-β pathway activation and, thus, immune evasion (90, 91). Another pleiotropic cytokine, IL-6, secreted from TAM, induces and maintains the CSC phenotype via STAT3 signaling (92–94). Hypoxia induces the CSC phenotype in BC cells by activating the NOTCH1 signaling pathway (94, 95), while CCL20 induces the BCSC phenotype via the NF-κB pathway (90, 93, 94, 96). PD-L1, an important checkpoint molecule, is positively correlated with BCSC expression (97, 98). Thus, BCSCs employ a variety of mechanisms to bypass antitumor immunity. Therefore, targeting these CSCs to restore the host immunity will be an effective therapeutic strategy.

The transcription factors OCT4, SOX2, NANOG, BMI1, and KLF4 are associated with the stemness and aggressiveness of BC. OCT4 and SOX2 are associated with high histologic grade tumors, whereas NANOG, BMI1, and KLF4 are common in low histologic grade tumors and hormone receptor-positive BCs (99). The expression of these transcription factors varies among different molecular subtypes of BC. The expression of OCT4 and SOX2 is the lowest in the luminal A subtype. However, luminal A and luminal B subtypes have a higher expression of NANOG, BMI1, and KLF4 than the HER2 and TNBC subtypes (99). Stemness and EMT are two correlated events. OCT4 is an important inducer of EMT (100). In lung cancer, it upregulates the expression of mesenchymal markers like vimentin and N-cadherin and promotes the degradation of the β-catenin/E-cadherin complex, thereby facilitating the invasion of the CSCs (100, 101). However, in breast tumors, the result is quite contrasting. In a study conducted by Hu et al., it was shown that silencing OCT4 induced EMT and promoted the invasion of the human breast cancer cell line MCF7 (101). Like OCT4, SOX2 is also associated with tumor aggressiveness, whereas NANOG is associated with lymph node metastasis of breast tumors (99–103).

## 5 CSC targeted therapy in breast cancer

In recent years, the development of molecular subtype-specific therapies has significantly increased the overall survival rate (ORR) for BC patients, particularly with metastatic spread. These therapies principally encompass third-generation hormonal therapies against hormone-susceptive BC, HER2-targeting agents against HER2-overexpressing BC, and PARP inhibitors to counter TNBC (104). Despite these advancements, many patients still experience relapse. Accumulating pieces of evidence suggest that a very small malignant subpopulation with stem cell properties residing within BC contributes to these phenomena. On account of their relative resistance to conventional therapies and tumorigenic potency, these BCSCs persist against genotoxic hits and contribute to treatment resistance and induce relapse. That is why framing treatment strategies against BCSCs is urgently needed.

BCSCs being a very small subset within a malignant bulk require proper identification for therapeutic targeting. The development of biomarkers to identify BCSCs has fast-tracked such as identification and characterization. The BCSC marker CD44 has been indicated as a biomarker for diagnostic, therapeutic, and prognostic purposes (**Table 1**). A significant decrease in sphere formation and tumor growth in severe combined immunodeficiency (SCID) mice was observed after the knockdown of CD44 (110). Various CD44 targeting avenues include CD44-specific antibodies, competitive proteins saturating CD44-binding sites, chemotherapy agents, and CD44-siRNA coupled to CD44-binding partners such as hyaluronic acid (108). The biomarker CD133 (prominin-1) is linked with high tumorigenicity and spheroid-forming ability (109). The therapeutic approaches to target CD133 govern antibody-based targeting and CAR T-cell therapy (clinical trial no. NCT02541370) (6, 111) (**Table 1**). In BC, epithelial cellular adhesion molecule (EpCAM) expression positively correlates to CSC-like phenotypes which further promote bone metastasis (112). Targeting EpCAM^+^ BCSCs (**Table 1**) with multiple antibody formats including EpCAM toxin-conjugated antibodies like oportuzumab monatox [scFv antibody and Pseudomonas exotoxin A (ETA)], citatuzumab bogatox (Fab fragment with bouganin toxin), and immuno-conjugate antibody tucotuzumab (monoclonal antibody with IL-2) have yielded positive response (113).

**Table 1:** List of therapeutic agents targeting BCSCs.

Sl. no.	Target	Therapeutic agents
1.	CD44	CD44-specific antibodies, chemotherapy agents, and CD44-siRNA coupled to CD44-binding partners like hyaluronic acid (100)
2.	CD133	Antibody-based targeting and CAR T-cell therapy (102, 103)
3.	EpCAM	Oportuzumab monatox, citatuzumab bogatox, tucotuzumab (105)
4.	NOTCH Signaling	GSI alone or in combination with therapeutic agents like docetaxel, paclitaxel, and carboplatin (106)
5.	Hedgehog signaling	SMO inhibitors like vismodegib and sonidegib and GLI1 inhibitors like GANT58 and GANT61 (107)
6.	Drug efflux pump inhibitors	ABC inhibitors like pheophorbide (ABCG2 inhibitor) and camptothecin (ABCC1 inhibitor) (108, 109)

The second approach to counter BCSCs is to target signaling networks that govern the CSC phenotype and functionality. In BC, the NOTCH, WNT/β-catenin, and Hedgehog (Hh) pathways (**Table 1**) regulate the self-renewal of BCSCs. Aberrant activation of the NOTCH signaling pathway promoting chemo-/radioresistance in BSCSs is prevented by gamma-secretase inhibitors (GSI). GSI administered alone or in combination with chemotherapeutic agents like docetaxel (GSI MK-0752), paclitaxel, and carboplatin has proven its efficacy and currently undergoing clinical trial (114).

The WNT/β-catenin pathway regulates not only BCSC maintenance but also cellular plasticity through EMT modulation (115). Aberrant activation of WNT has been neutralized by WNT inhibitors, which are now entering phase I clinical trials (116, 117). Hh signaling proteins like SMO and GLI1 selectively upregulate BCSCs, particularly in TNBC through paracrine secretion. SMO inhibitors like vismodegib and sonidegib are passing through clinical trials for basal cell carcinoma. Additionally, clinical trials are now focusing on the combination therapy of SMO inhibitors and chemotherapeutic drugs in TNBC. GLI1 inhibitors like GANT58 and GANT61 are also under preclinical drug development (118). Several studies have also reported that HER2-signaling-regulated BCSC activities, including self-renewal and radioresistance, have been counteracted through HER2-specific antibodies like trastuzumab, which is going through a phase III clinical trial (119, 120).

Another outlook in BCSC targeting is neutralizing their overexpressed drug efflux pumps like ABC transporters. Because of their strong drug efflux properties, they serve as a strong protective mechanism against xenobiotic altercations, particularly during conventional chemotherapies. Utilizing ABC inhibitors like pheophorbide (ABCG2 inhibitor) and camptothecin (ABCC1 inhibitor), BCSCs are sensitized (121, 122).

Another perspective that is employed in BCSC targeting is immunotherapy. Immunotherapy targets BCSCs by modulating immune cells such as cytokine-induced killer (CIK) cells, NK cells, γδ T cells, and CD8^+^ T cells and through DC-based vaccines (123, 124). Adoptive transfer of reinfused TILs, e.g., CAR T (chimeric antigen receptor T) cells or CIK cells, has shown therapeutic efficacy. Adoptive transfer of CD8^+^ T cells and γδ cells has elicited antigen-specific killing by CD8^+^ T cells in BCSCs (71). Very often, immunotherapies are used in combination with chemotherapy, viz., atezolizumab (a PD-L1 inhibitor) coupled with albumin-bound paclitaxel to treat metastatic TNBC (125, 126). BCSCs also exhibit selective upregulation of various cancer testis (CT) antigens like MAGEA12, PA17, PLU-1, and TEX15 (127). Since these antigens are normally expressed only in germline cells, which remain devoid of MHC molecules, immune targeting via CT-specific T cells appears to be promising. So far, MAGE-A and NY-ESO-1-based vaccines are currently under clinical trials (127).

Targeting BCSC markers like ALDH1, CD44, CD133, and CD24 with specific agents and antibodies combined with chemotherapy can achieve a good therapeutic effect (6). Positive results were obtained by combining anti-CD44 antibody and gemcitabine with magnetic nanoparticles (MNPs) (105). Similarly, low-dose decitabine and doxorubicin along with nanoparticles yielded better results in ALDH^high^ CSCs (105). Normal stem cells with many different signaling pathways can be turned into CSCs. Signaling pathways such as TGF-β, NOTCH, JAK-STAT, WNT, Hh, and NF-κB regulate the survivability of CSCs. Ligand antibodies and GSI are found to inhibit the NOTCH signaling pathways. MK-0752 along with docetaxel is found suitable for the treatment of BC. Targeting the DLL4 protein (an angiogenic regulator) can also block the NOTCH pathway. CSC stemness was also regulated by smoothened (SMO) protein through Hh signaling. Sonidegib (an antagonist of SMO) with docetaxel is mainly used to treat a TNBC patient. WNT induces EMT and cellular plasticity leading to the generation of CSCs. Hence, WNT inhibitors restrained the growth of BCSCs, and their effect was prominent on both *in-vitro* and xenograft models. Targeting the HER2 signaling pathways is very successful in BC evasion. Lapatinib and trastuzumab and HER2 targeted antibodies increased patient survival with advanced stage ER^+^ cancer, reducing the CSC population. Lapatinib reduced the CD44^+^/CD24^low^ population and the self-renewing ability, and this was found through mammosphere formation (106). Treating antibodies against the IL-8 receptor CXCR1 and repertaxin (CXCR1/CXCR2 inhibitor) targets BCSCs in xenograft models, thus reducing the EMT and tumor growth.

Immunotherapy is also a potential strategy to counteract tumor growth and recurrence. It was reported that chimeric antigen receptor therapy T (CAR T) is a successful therapy for leukemia, so these treatments can also be examined in BCSC therapy. Reports suggest that DC vaccines elicit a better immune response by increasing the stimulation of interferon-γ expression. Blocking T-cell regulation by targeting PDL, PDL-1, and CTLA-4 showed a positive response in BCSC treatment. The FDA has approved a combined therapy of atezolizumab (a PD-L1 inhibitor) along with albumin-bound paclitaxel to treat metastatic TNBC (107, 128–130). Consequent *in-vitro* upregulation of PD-L1 in BCSCs has been reported, which however needs to be validated by further studies (107, 129, 130).

## 6 Conclusion and future direction

The heterogeneity of the cellular population and their function and in some cases the plasticity of the phenotypic behavior in TME plays a key role in determining the evolution and fate of BC. The complex network of interactions between BCSCs and various immune cells impacts heavily on tumor initiation as well as the metastatic behavior of BC. These modulated TME components signal for the acquisition and/or promotion of BCSC traits. This interaction also encourages EMT traits in the local and distant sites, thereby spreading the cancer. Understanding the molecular mechanism of such interaction will definitely help in preparing tumor-eradicating therapy not only by reducing tumor but also by eliminating future possibilities of recurrence and metastasis. Pointing out of these overlooked areas will help control BC in better non-conventional ways.

## Author contributions

SBa, AG, and KG conceived and planned the article. AG, JS, PC, MC, KG, and SBa wrote the manuscript. NG prepared the graphical illustration. ABh, AS, SBe, SD, JD, TD, RB, and ABo edited the manuscript. All authors contributed to the article and approved the submitted version.

## References

[1] Yin L Duan JJ Bian XW Yu SC. Triple-negative breast cancer molecular subtyping and treatment progress. Breast Cancer Res (2020) 22(1):1–3. doi: 10.1186/s13058-020-01296-5 PMC7285581 32517735

[2] Lehmann BD Bauer JA Chen X Sanders ME Chakravarthy AB Shyr Y. Identification of human triple-negative breast cancer subtypes and preclinical models for selection of targeted therapies. J Clin Invest (2011) 121(7):2750–67. doi: 10.1172/JCI45014 PMC3127435 21633166

[3] Crabtree JS Miele L. Breast cancer stem cells. Biomedicines. (2018) 6(3):77. doi: 10.3390/biomedicines6030077 30018256 PMC6163894

[4] Lagadec C Vlashi E Della Donna L Dekmezian C Pajonk F. Radiation-induced reprogramming of breast cancer cells. Stem Cells (2012) 30(5):833–44. doi: 10.1002/stem.1058 PMC3413333 22489015

[5] Rios AC Fu NY Lindeman GJ Visvader JE. *In situ* identification of bipotent stem cells in the mammary gland. Nature. (2014) 506(7488):322–7. doi: 10.1038/nature12948 24463516

[6] Walcher L Kistenmacher AK Suo H Kitte R Dluczek S Strauß A. Cancer stem cells—origins and biomarkers: perspectives for targeted personalized therapies. Front Immunol (2020) 11:1280. doi: 10.3389/fimmu.2020.01280 32849491 PMC7426526

[7] Sin WC Lim CL. Breast cancer stem cells—from origins to targeted therapy. Stem Cell Invest (2017) 4. doi: 10.21037/sci.2017.11.03 PMC5723743 29270422

[8] Luo M Clouthier SG Deol Y Liu S Nagrath S Azizi E. Breast cancer stem cells: current advances and clinical implications. Mammary Stem Cells (2015) 1293:1–49. doi: 10.1007/978-1-4939-2519-3_1 26040679

[9] Zhou J Chen Q Zou Y Chen H Qi L Chen Y. Stem cells and cellular origins of breast cancer: updates in the rationale, controversies, and therapeutic implications. Front Oncol (2019) 9:820. doi: 10.3389/fonc.2019.00820 31555586 PMC6722475

[10] Khoury T Ademuyiwa FO Chandraseekhar R Jabbour M DeLeo A Ferrone S. Aldehyde dehydrogenase 1A1 expression in breast cancer is associated with stage, triple negativity, and outcome to neoadjuvant chemotherapy. Modern Pathology (2012) 25(3):388–97. doi: 10.1038/modpathol.2011.172 PMC3426278 22080062

[11] Wei Y Li Y Chen Y Liu P Huang S Zhang Y. ALDH1: A potential therapeutic target for cancer stem cells in solid tumors. Front Oncol (2022) 12:1026278. doi: 10.3389/fonc.2022.1026278 36387165 PMC9650078

[12] Liu M Liu Y Deng L Wang D He X Zhou L. Transcriptional profiles of different states of cancer stem cells in triple-negative breast cancer. Mol canc (2018) 17(1):1–6. doi: 10.1186/s12943-018-0809-x PMC5824475 29471829

[13] Yu KD Zhu R Zhan M Rodriguez AA Yang W Wong S. Identification of prognosis-relevant subgroups in patients with chemoresistant triple-negative breast cancer. Clin Cancer Res (2013) 19(10):2723–33. doi: 10.1158/1078-0432.CCR-12-2986 PMC3655097 23549873

[14] Park SY Choi JH Nam JS. Targeting cancer stem cells in triple-negative breast cancer. Cancers. (2019) 11(7):965. doi: 10.3390/cancers11070965 31324052 PMC6678244

[15] Liu S Cong Y Wang D Sun Y Deng L Liu Y. Breast cancer stem cells transition between epithelial and mesenchymal states reflective of their normal counterparts. Stem Cell Rep (2014) 2(1):78–91. doi: 10.1016/j.stemcr.2013.11.009 PMC3916760 24511467

[16] Brabletz T. EMT. and MET in metastasis: where are the cancer stem cells? Cancer Cell (2012) 22(6):699–701. doi: 10.1016/j.ccr.2012.11.009 23238008

[17] Palomeras S Ruiz-Martínez S Puig T. Targeting breast cancer stem cells to overcome treatment resistance. Molecules. (2018) 23(9):2193. doi: 10.3390/molecules23092193 30200262 PMC6225226

[18] Abel AM Yang C Thakar MS Malarkannan S. Natural killer cells: development, maturation, and clinical utilization. Front Immunol (2018) 9:1869. doi: 10.3389/fimmu.2018.01869 30150991 PMC6099181

[19] Vivier E Raulet DH Moretta A Caligiuri MA Zitvogel L Lanier LL. Innate or adaptive immunity The example of natural killer cells. science. (2011) 331(6013):44–9. doi: 10.1126/science.1198687 PMC3089969 21212348

[20] Melaiu O Lucarini V Cifaldi L Fruci D. Influence of the tumor microenvironment on NK cell function in solid tumors. Front Immunol (2020) 10:3038. doi: 10.3389/fimmu.2019.03038 32038612 PMC6985149

[21] Wang B Wang Q Wang Z Jiang J Yu SC Ping YF. Metastatic consequences of immune escape from NK cell cytotoxicity by human breast cancer stem cells. Cancer Res (2014) 74(20):5746–57. doi: 10.1158/0008-5472.CAN-13-2563 25164008

[22] Rowley DA Fitch FW. The road to the discovery of dendritic cells, a tribute to Ralph Steinman. Cell Immunol (2012) 273(2):95–8. doi: 10.1016/j.cellimm.2012.01.002 22326169

[23] Sharma M Hegde P Aimanianda V Beau R Maddur MS Sénéchal H. Circulating human basophils lack the features of professional antigen presenting cells. Sci Rep (2013) 3(1):1–8. doi: 10.1038/srep01188 PMC3561623 23378919

[24] Kim MK Kim J. Properties of immature and mature dendritic cells: phenotype, morphology, phagocytosis, and migration. RSC advances (2019) 9(20):11230–8. doi: 10.1039/C9RA00818G PMC9063012 35520256

[25] Alloatti A Kotsias F Magalhaes JG Amigorena S. Dendritic cell maturation and cross-presentation: timing matters! Immunol Rev (2016) 272(1):97–108. doi: 10.1111/imr.12432 27319345 PMC6680313

[26] Verneau J Sautés-Fridman C Sun CM. Dendritic cells in the tumor microenvironment: prognostic and theranostic impact. In: Seminars in Immunology. Academic Press: Elsevier (2020) 48, 101410. 33011065 10.1016/j.smim.2020.101410

[27] Harizi H. Reciprocal crosstalk between dendritic cells and natural killer cells under the effects of PGE2 in immunity and immunopathology. Cell Mol Immunol (2013) 10(3):213–21. doi: 10.1038/cmi.2013.1 PMC4012770 23524652

[28] Van Elssen CH Vanderlocht J Oth T Senden-Gijsbers BL Germeraad WT Bos GM. Inflammation restraining effects of prostaglandin E2 on natural killer–dendritic cell (NK-DC) interaction are imprinted during DC maturation. Blood J Am Soc Hematology (2011) 118(9):2473–82. doi: 10.1182/blood-2010-09-307835 21715307

[29] Aramini B Masciale V Grisendi G Banchelli F D’Amico R Maiorana A. Cancer stem cells and macrophages: molecular connections and future perspectives against cancer. Oncotarget. (2021) 12(3):230. doi: 10.18632/oncotarget.27870 33613850 PMC7869576

[30] Gutknecht MF Bouton AH. Functional significance of mononuclear phagocyte populations generated through adult hematopoiesis. J leukocyte Biol (2014) 96(6):969–80. doi: 10.1189/jlb.1RI0414-195R PMC4226790 25225678

[31] Guo Q Jin Z Yuan Y Liu R Xu T Wei H. Corrigendum to “new mechanisms of tumor-associated macrophages on promoting tumor progression: recent research advances and potential targets for tumor immunotherapy”. J Immunol Res (2018) 2018. doi: 10.1155/2018/6728474 PMC6087574 30151395

[32] Ge Z Ding S. The crosstalk between tumor-associated macrophages (TAMs) and tumor cells and the corresponding targeted therapy. Front Oncol (2020) 10:2404. doi: 10.3389/fonc.2020.590941 PMC7670061 33224886

[33] Velasco-Velázquez MA Popov VM Lisanti MP Pestell RG. The role of breast cancer stem cells in metastasis and therapeutic implications. Am J pathology (2011) 179(1):2–11. doi: 10.1016/j.ajpath.2011.03.005 PMC3123864 21640330

[34] Yang C He L He P Liu Y Wang W He Y. Increased drug resistance in breast cancer by tumor-associated macrophages through IL-10/STAT3/bcl-2 signaling pathway. Med Oncol (2015) 32(2):14. doi: 10.1007/s12032-014-0352-6 25572805

[35] Wan S Zhao E Kryczek I Vatan L Sadovskaya A Ludema G. Tumor-associated macrophages produce interleukin 6 and signal via STAT3 to promote expansion of human hepatocellular carcinoma stem cells. Gastroenterology. (2014) 147(6):1393–404. doi: 10.1053/j.gastro.2014.08.039 PMC4253315 25181692

[36] Bonavita E Galdiero MR Jaillon S Mantovani A. Phagocytes as corrupted policemen in cancer-related inflammation. Adv Cancer Res (2015) 128:141–71. doi: 10.1016/bs.acr.2015.04.013 26216632

[37] Zhou N Zhang Y Zhang X Lei Z Hu R Li H. Exposure of tumor-associated macrophages to apoptotic MCF-7 cells promotes breast cancer growth and metastasis. Int J Mol Sci (2015) 16(6):11966–82. doi: 10.3390/ijms160611966 PMC4490423 26016502

[38] Hegde S Leader AM Merad M. MDSC: Markers, development, states, and unaddressed complexity. Immunity. (2021) 54(5):875–84. doi: 10.1016/j.immuni.2021.04.004 PMC8709560 33979585

[39] Veglia F Sanseviero E Gabrilovich DI. Myeloid-derived suppressor cells in the era of increasing myeloid cell diversity. Nat Rev Immunol (2021) 21(8):485–98. doi: 10.1038/s41577-020-00490-y PMC7849958 33526920

[40] Ribatti D Tamma R Annese T. Epithelial-mesenchymal transition in cancer: a historical overview. Trans Oncol (2020) 13(6):100773. doi: 10.1016/j.tranon.2020.100773 PMC7182759 32334405

[41] Huang Y Hong W Wei X. The molecular mechanisms and therapeutic strategies of EMT in tumor progression and metastasis. J Hematol Oncol (2022) 15(1):129. doi: 10.1186/s13045-022-01347-8 36076302 PMC9461252

[42] Sainz B Carron E Vallespinós M MaChado HL. Cancer stem cells and macrophages: implications in tumor biology and therapeutic strategies. Mediators inflammation (2016) 2016. doi: 10.1155/2016/9012369 PMC4769767 26980947

[43] Peng D Tanikawa T Li W Zhao L Vatan L Szeliga W. Myeloid-derived suppressor cells endow stem-like qualities to breast cancer cells through IL6/STAT3 and NO/NOTCH cross-talk signaling. Cancer Res (2016) 76(11):3156–65. doi: 10.1158/0008-5472.CAN-15-2528 PMC4891237 27197152

[44] Janghorban M Xin L Rosen JM Zhang XH. Notch signaling as a regulator of the tumor immune response: to target or not to target? Front Immunol (2018) 9:1649. doi: 10.3389/fimmu.2018.01649 30061899 PMC6055003

[45] Kumar S Wilkes DW Samuel N Blanco MA Nayak A Alicea-Torres K. ΔNp63-driven recruitment of myeloid-derived suppressor cells promotes metastasis in triple-negative breast cancer. J Clin Invest (2018) 128(11):5095–109. doi: 10.1172/JCI99673 PMC6205409 30295647

[46] Mukaida N Sasaki SI Baba T. Two-faced roles of tumor-associated neutrophils in cancer development and progression. Int J Mol Sci (2020) 21(10):3457. doi: 10.3390/ijms21103457 32422991 PMC7278934

[47] Peltanova B Raudenska M Masarik M. Effect of tumor microenvironment on pathogenesis of the head and neck squamous cell carcinoma: a systematic review. Mol canc (2019) 18(1):1–24. doi: 10.1186/s12943-019-0983-5 PMC6441173 30927923

[48] Liu C Huang Z Wang Q Sun B Ding L Meng X. Usefulness of neutrophil-to-lymphocyte ratio and platelet-to-lymphocyte ratio in hormone-receptor-negative breast cancer. OncoTargets Ther (2016) 9:4653. doi: 10.2147/OTT.S106017 PMC4973777 27536129

[49] Banyard J Bielenberg DR. The role of EMT and MET in cancer dissemination. Connective Tissue Res (2015) 56(5):403–13. doi: 10.3109/03008207.2015.1060970 PMC4780319 26291767

[50] SenGupta S Hein LE Xu Y Zhang J Konwerski JR Li Y. Triple-negative breast cancer cells recruit neutrophils by secreting TGF-β and CXCR2 ligands. Front Immunol (2021) 12:973. doi: 10.3389/fimmu.2021.659996 PMC8071875 33912188

[51] Varricchi G Galdiero MR Loffredo S Lucarini V Marone G Mattei F. Eosinophils: The unsung heroes in cancer? Oncoimmunology. (2018) 7(2):e1393134. doi: 10.1080/2162402X.2017.1393134 29308325 PMC5749653

[52] Krystel-Whittemore M Dileepan KN Wood JG. Mast cell: a multi-functional master cell. Front Immunol (2016) 620. doi: 10.3389/fimmu.2015.00620 PMC4701915 26779180

[53] Kokubo K Onodera A Kiuchi M Tsuji K Hirahara K Nakayama T. Conventional and pathogenic Th2 cells in inflammation, tissue repair, and fibrosis. Front Immunol (2022) 13:945063. doi: 10.3389/fimmu.2022.945063 36016937 PMC9395650

[54] Carmeliet P. VEGF as a key mediator of angiogenesis in cancer. Oncology. (2005) 69(Suppl. 3):4–10. doi: 10.1159/000088478 16301830

[55] Liu KW Hu B Cheng SY. Platelet-derived growth factor signaling in human Malignancies. Chin J canc (2011) 30(9):581. doi: 10.5732/cjc.011.10300 PMC3525704 21880178

[56] Korc M Friesel RE. The role of fibroblast growth factors in tumor growth. Curr Cancer Drug targets (2009) 9(5):639–51. doi: 10.2174/156800909789057006 PMC3664927 19508171

[57] Turner N Grose R. Fibroblast growth factor signalling: from development to cancer. Nat Rev Canc (2010) 10(2):116–29. doi: 10.1038/nrc2780 20094046

[58] Lucarini V Ziccheddu G Macchia I La Sorsa V Peschiaroli F Buccione C. IL-33 restricts tumor growth and inhibits pulmonary metastasis in melanoma-bearing mice through eosinophils. Oncoimmunology. (2017) 6(6):e1317420. doi: 10.1080/2162402X.2017.1317420 28680750 PMC5486175

[59] Chakravarti M Dhar S Bera S Sinha A Roy K Sarkar A. Terminally exhausted CD8+ T cells resistant to PD-1 blockade promote generation and maintenance of aggressive cancer stem cells. Cancer Res (2023) 83(11):1815–33. doi: 10.1158/0008-5472.CAN-22-3864 36971604

[60] Riaz N Havel JJ Makarov V Desrichard A Urba WJ Sims JS. Tumor and microenvironment evolution during immunotherapy with nivolumab. Cell. (2017) 171(4):934–49. doi: 10.1016/j.cell.2017.09.028 PMC5685550 29033130

[61] Narang P Chen M Sharma AA Anderson KS Wilson MA. The neoepitope landscape of breast cancer: implications for immunotherapy. BMC canc (2019) 19(1):1–0. doi: 10.1186/s12885-019-5402-1 PMC6399957 30832597

[62] Macías-Paz IU Rivera-Arenas A Reyna-Beltrán E Tavera-Tapia A. New insights about organoids as model of study for breast cancer research. Gaceta mexicana oncología (2022) 21(4):135–42. doi: 10.24875/j.gamo.22000110

[63] Yalcin GD Danisik N Baygin RC Acar A. Systems biology and experimental model systems of cancer. J Personalized Med (2020) 10(4):180. doi: 10.3390/jpm10040180 PMC7712848 33086677

[64] Sachs N de Ligt J Kopper O Gogola E Bounova G Weeber F. A living biobank of breast cancer organoids captures disease heterogeneity. Cell. (2018) 172(1):373–86. doi: 10.1016/j.cell.2017.11.010 29224780

[65] Seo AN Lee HJ Kim EJ Kim HJ Jang MH Lee HE. Tumour-infiltrating CD8+ lymphocytes as an independent predictive factor for pathological complete response to primary systemic therapy in breast cancer. Br J canc (2013) 109(10):2705–13. doi: 10.1038/bjc.2013.634 PMC3833219 24129232

[66] Pellegrino B Hlavata Z Migali C De Silva P Aiello M Willard-Gallo K. Luminal breast cancer: Risk of recurrence and tumor-associated immune suppression. Mol Diagnosis Ther (2021) 25(4):409–24. doi: 10.1007/s40291-021-00525-7 PMC8249273 33974235

[67] Müller L Tunger A Plesca I Wehner R Temme A Westphal D. Bidirectional crosstalk between cancer stem cells and immune cell subsets. Front Immunol (2020) 11:140. doi: 10.3389/fimmu.2020.00140 32117287 PMC7013084

[68] Stapelberg M Zobalova R Nguyen MN Walker T Stantic M Goodwin J. Indoleamine-2, 3-dioxygenase elevated in tumor-initiating cells is suppressed by mitocans. Free Radical Biol Med (2014) 67:41–50. doi: 10.1016/j.freeradbiomed.2013.10.003 24145120

[69] Prendergast GC Smith C Thomas S Mandik-Nayak L Laury-Kleintop L Metz R. Indoleamine 2, 3-dioxygenase pathways of pathogenic inflammation and immune escape in cancer. Cancer immunology Immunother (2014) 63(7):721–35. doi: 10.1007/s00262-014-1549-4 PMC4384696 24711084

[70] Xu Y Dong X Qi P Ye Y Shen W Leng L. Sox2 communicates with tregs through CCL1 to promote the stemness property of breast cancer cells. Stem Cells (2017) 35(12):2351–65. doi: 10.1002/stem.2720 PMC5958902 29044882

[71] Chen HC Joalland N Bridgeman JS Alchami FS Jarry U Khan MW. Synergistic targeting of breast cancer stem-like cells by human γδ T cells and CD8+ T cells. Immunol Cell Biol (2017) 95(7):620–9. doi: 10.1038/icb.2017.21 PMC5550559 28356569

[72] Holinstat M. Normal platelet function. Cancer Metastasis Rev (2017) 36(2):195–8. doi: 10.1007/s10555-017-9677-x PMC5709181 28667366

[73] Mounce LT Hamilton W Bailey SE. Cancer incidence following a high-normal platelet count: cohort study using electronic healthcare records from English primary care. Br J Gen Practice. (2020) 70(698):e622–8. doi: 10.3399/bjgp20X710957 PMC7390285 32719013

[74] Liu S Fang J Jiao D Liu Z. Elevated platelet count predicts poor prognosis in breast cancer patients with supraclavicular lymph node metastasis. Cancer Manage Res (2020) 12:6069. doi: 10.2147/CMAR.S257727 PMC7381764 32765104

[75] Moskalensky AE Yurkin MA Muliukov AR Litvinenko AL Nekrasov VM Chernyshev AV. Method for the simulation of blood platelet shape and its evolution during activation. PloS Comput Biol (2018) 14(3):e1005899. doi: 10.1371/journal.pcbi.1005899 29518073 PMC5860797

[76] Zuo XX Yang Y Zhang Y Zhang ZG Wang XF Shi YG. Platelets promote breast cancer cell MCF-7 metastasis by direct interaction: surface integrin α2β1-contacting-mediated activation of Wnt-β-catenin pathway. Cell Communication Signaling (2019) 17(1):1–5. doi: 10.1186/s12964-019-0464-x 31699102 PMC6836423

[77] Wang S Li Z Xu R. Human cancer and platelet interaction, a potential therapeutic target. Int J Mol Sci (2018) 19(4):1246. doi: 10.3390/ijms19041246 29677116 PMC5979598

[78] Schmied L Höglund P Meinke S. Platelet-mediated protection of cancer cells from immune surveillance–possible implications for cancer immunotherapy. Front Immunol (2021) 12:527. doi: 10.3389/fimmu.2021.640578 PMC7988080 33777033

[79] Huang TX Guan XY Fu L. Therapeutic targeting of the crosstalk between cancer-associated fibroblasts and cancer stem cells. Am J Cancer Res (2019) 9(9):1889. 31598393 PMC6780671

[80] Polanska UM Acar A Orimo A. Experimental generation of carcinoma-associated fibroblasts (CAFs) from human mammary fibroblasts. JoVE (Journal Visualized Experiments) (2011) 25(56):e3201. doi: 10.3791/3201 PMC3227206 22064505

[81] Chan TS Shaked Y Tsai KK. Targeting the interplay between cancer fibroblasts, mesenchymal stem cells, and cancer stem cells in desmoplastic cancers. Front Oncol (2019) 9:688. doi: 10.3389/fonc.2019.00688 31417869 PMC6684765

[82] Huang M Li Y Zhang H Nan F. Breast cancer stromal fibroblasts promote the generation of CD44+ CD24-cells through SDF-1/CXCR4 interaction. J Exp Clin Cancer Res (2010) 29(1):1–0. doi: 10.1186/1756-9966-29-80 20569497 PMC2911413

[83] Shuo W Ying Z Weihong C Jie L Yuren Z Huiting F. Breast cancer stem-like cells can promote metastasis by activating platelets and down-regulating antitumor activity of natural killer cells. J Traditional Chin Med (2016) 36(4):530–7. doi: 10.1016/S0254-6272(16)30071-1 28459521

[84] Sultan M Vidovic D Paine AS Huynh TT Coyle KM Thomas ML. Epigenetic silencing of TAP1 in Aldefluor+ breast cancer stem cells contributes to their enhanced immune evasion. Stem Cells (2018) 36(5):641–54. doi: 10.1002/stem.2780 29341428

[85] Lazarova M Steinle A. Impairment of NKG2D-mediated tumor immunity by TGF-β. Front Immunol (2019) 10:2689. doi: 10.3389/fimmu.2019.02689 31803194 PMC6873348

[86] Vinay DS Ryan EP Pawelec G Talib WH Stagg J Elkord E. Immune evasion in cancer: Mechanistic basis and therapeutic strategies. In: Seminars in cancer biology, Academic Press: Elsevier (2015). vol. 35, S185–98. 10.1016/j.semcancer.2015.03.004 25818339

[87] Tsuchiya H Shiota G. Immune evasion by cancer stem cells. Regenerative Ther (2021) 17:20–33. doi: 10.1016/j.reth.2021.02.006 PMC7966825 33778133

[88] Lauricella M Carlisi D Giuliano M Calvaruso G Cernigliaro C Vento R. The analysis of estrogen receptor-α positive breast cancer stem-like cells unveils a high expression of the serpin proteinase inhibitor PI-9: Possible regulatory mechanisms. Int J Oncol (2016) 49(1):352–60. doi: 10.3892/ijo.2016.3495 27121069

[89] Espinoza-Sánchez NA Vadillo E Balandrán JC Monroy-García A Pelayo R Fuentes-Pananá EM. Evidence of lateral transmission of aggressive features between different types of breast cancer cells. Int J Oncol (2017) 51(5):1482–96. doi: 10.3892/ijo.2017.4128 PMC5643070 29048610

[90] AlHossiny M Luo L Frazier WR Steiner N Gusev Y Kallakury B. Ly6E/K signaling to TGFβ promotes breast cancer progression, immune escape, and drug resistance. Cancer Res (2016) 76(11):3376–86. doi: 10.1158/0008-5472.CAN-15-2654 PMC4910623 27197181

[91] Shipitsin M Campbell LL Argani P Weremowicz S Bloushtain-Qimron N Yao J. Molecular definition of breast tumor heterogeneity. Cancer Cell (2007) 11(3):259–73. doi: 10.1016/j.ccr.2007.01.013 17349583

[92] Chng Z Teo A Pedersen RA Vallier L. SIP1 mediates cell-fate decisions between neuroectoderm and mesendoderm in human pluripotent stem cells. Cell Stem Cell (2010) 6(1):59–70. doi: 10.1016/j.stem.2009.11.015 20074535

[93] Korkaya H Liu S Wicha MS. Breast cancer stem cells, cytokine networks, and the tumor microenvironment. J Clin Invest (2011) 121(10):3804–9. doi: 10.1172/JCI57099 PMC3223613 21965337

[94] Balamurugan K Mendoza-Villanueva D Sharan S Summers GH Dobrolecki LE Lewis MT. C/EBPδ links IL-6 and HIF-1 signaling to promote breast cancer stem cell-associated phenotypes. Oncogene. (2019) 38(20):3765–80. doi: 10.1038/s41388-018-0516-5 PMC6437025 30262865

[95] Lottaz C Beier D Meyer K Kumar P Hermann A Schwarz J. Transcriptional profiles of CD133+ and CD133– glioblastoma-derived cancer stem cell lines suggest different cells of origin. Cancer Res (2010) 70(5):2030–40. doi: 10.1158/0008-5472.CAN-09-1707 20145155

[96] Chen W Qin Y Wang D Zhou L Liu Y Chen S. CCL20 triggered by chemotherapy hinders the therapeutic efficacy of breast cancer. PloS Biol (2018) 16(7):e2005869. doi: 10.1371/journal.pbio.2005869 30052635 PMC6082578

[97] Polónia A Pinto R Cameselle-Teijeiro JF Schmitt FC Paredes J. Prognostic value of stromal tumour infiltrating lymphocytes and programmed cell death-ligand 1 expression in breast cancer. J Clin pathology (2017) 70(10):860–7. doi: 10.1136/jclinpath-2016-203990 28373294

[98] Inaguma S Lasota J Wang Z Czapiewski P Langfort R Rys J. Expression of ALCAM (CD166) and PD-L1 (CD274) independently predicts shorter survival in Malignant pleural mesothelioma. Hum pathology (2018) 71:1–7. doi: 10.1016/j.humpath.2017.04.032 PMC5748003 28811252

[99] Gwak JM Kim M Kim HJ Jang MH Park SY. Expression of embryonal stem cell transcription factors in breast cancer: Oct4 as an indicator for poor clinical outcome and tamoxifen resistance. Oncotarget. (2017) 8(22):36305. doi: 10.18632/oncotarget.16750 28422735 PMC5482656

[100] Chen ZS Ling DJ Zhang YD Feng JX Zhang XY Shi TS. Octamer-binding protein 4 affects the cell biology and phenotypic transition of lung cancer cells involving β-catenin/E-cadherin complex degradation. Mol Med Rep (2015) 11(3):1851–8. doi: 10.3892/mmr.2014.2992 25420671

[101] Hu J Qin K Zhang Y Gong J Li N Lv D. Downregulation of transcription factor Oct4 induces an epithelial-to-mesenchymal transition via enhancement of Ca2+ influx in breast cancer cells. Biochem Biophys Res Commun (2011) 411(4):786–91. doi: 10.1016/j.bbrc.2011.07.025 21798248

[102] Lu X Mazur SJ Lin T Appella E Xu Y. The pluripotency factor nanog promotes breast cancer tumorigenesis and metastasis. Oncogene. (2014) 33(20):2655–64. doi: 10.1038/onc.2013.209 PMC3925756 23770853

[103] Wang D Lu P Zhang H Luo M Zhang X Wei X. Oct-4 and Nanog promote the epithelial-mesenchymal transition of breast cancer stem cells and are associated with poor prognosis in breast cancer patients. Oncotarget. (2014) 5(21):10803. doi: 10.18632/oncotarget.2506 25301732 PMC4279411

[104] Enzinger PC Burtness B Hollis D Niedzwiecki D Ilson D Benson AB. CALGB 80403/ECOG 1206: A randomized phase II study of three standard chemotherapy regimens (ECF, IC, FOLFOX) plus cetuximab in metastatic esophageal and GE junction cancer. J Clin Oncol (2010) 28(15_suppl):4006–. doi: 10.1200/jco.2010.28.15_suppl.4006

[105] Kargar PG Noorian M Chamani E Bagherzade G Kiani Z. Synthesis, characterization and cytotoxicity evaluation of a novel magnetic nanocomposite with iron oxide deposited on cellulose nanofibers with nickel (Fe 3 O 4@ NFC@ ONSM-Ni). RSC advances (2021) 11(28):17413–30. doi: 10.1039/D1RA01256H PMC9032764 35479678

[106] Ayob AZ Ramasamy TS. Cancer stem cells as key drivers of tumour progression. J Biomed science (2018) 25(1):1–8. doi: 10.1186/s12929-018-0426-4 PMC5838954 29506506

[107] Darvin P Sasidharan Nair V Elkord E. PD-L1 expression in human breast cancer stem cells is epigenetically regulated through posttranslational histone modifications. J Oncol (2019) 2019. doi: 10.1155/2019/3958908 PMC6409026 30915120

[108] Yang C He Y Zhang H Zhang H Liu Y Wang W Du Y. Selective killing of breast cancer cells expressing activated CD44 using CD44 ligand-coated nanoparticles in *vitro* and in vivo. Oncotarget (2015) 6(17):15283–96. doi: 10.18632/oncotarget.3681 PMC4558151 25909172

[109] Quigley HA Addicks EM. Chronic experimental glaucoma in primates. I. Production of elevated intraocular pressure by anterior chamber injection of autologous ghost red blood cells. Invest Ophthalmol Visual science (1980) 19(2):126–36. 6766124

[110] Nam K Oh S Lee KM Yoo SA Shin I. CD44 regulates cell proliferation, migration, and invasion via modulation of c-Src transcription in human breast cancer cells. Cell signalling (2015) 27(9):1882–94. doi: 10.1016/j.cellsig.2015.05.002 25979842

[111] Swaminathan SK Roger E Toti U Niu L Ohlfest JR Panyam J. CD133-targeted paclitaxel delivery inhibits local tumor recurrence in a mouse model of breast cancer. J Control Release (2013) 171(3):280–7. doi: 10.1016/j.jconrel.2013.07.014 23871962

[112] Hiraga T Ito S Nakamura H. EpCAM expression in breast cancer cells is associated with enhanced bone metastasis formation. Int J Canc (2016) 138(7):1698–708. doi: 10.1002/ijc.29921 26576938

[113] Eyvazi S Farajnia S Dastmalchi S Kanipour F Zarredar H Bandehpour M. Antibody based EpCAM targeted therapy of cancer, review and update. Curr Cancer Drug Targets (2018) 18(9):857–68. doi: 10.2174/1568009618666180102102311 29295696

[114] Schott AF Landis MD Dontu G Griffith KA Layman PM Krop I. Preclinical and clinical studies of gamma secretase inhibitors with docetaxel on human breast tumors. Clin Cancer Res (2013) 19(6):1512–24. doi: 10.1158/1078-0432.CCR-11-3326 PMC3602220 23340294

[115] Zhao C Blum J Chen A Kwon HY Jung SH Cook JM. Loss of β-catenin impairs the renewal of normal and CML stem cells in *vivo* . Cancer Cell (2007) 12(6):528–41. doi: 10.1016/j.ccr.2007.11.003 PMC2262869 18068630

[116] Gurney A Axelrod F Bond CJ Cain J Chartier C Donigan L. Wnt pathway inhibition via the targeting of Frizzled receptors results in decreased growth and tumorigenicity of human tumors. Proc Natl Acad Sci U S A (2012) 109(29):11717–22. doi: 10.1073/pnas.1120068109 PMC3406803 22753465

[117] Katoh M. Canonical and non-canonical WNT signaling in cancer stem cells and their niches: Cellular heterogeneity, omics reprogramming, targeted therapy and tumor plasticity. Int J Oncol (2017) 51(5):1357–69. doi: 10.3892/ijo.2017.4129 PMC5642388 29048660

[118] Bhateja P Cherian M Majumder S Ramaswamy B. The hedgehog signaling pathway: A viable target in breast cancer? Cancers (Basel) (2019) 11(8):1126. doi: 10.3390/cancers11081126 31394751 PMC6721501

[119] Mariani G Fasolo A De Benedictis E Gianni L. Trastuzumab as adjuvant systemic therapy for HER2-positive breast cancer. Nat Clin Pract Oncol (2009) 6(2):93–104. doi: 10.1038/ncponc1298 19107109

[120] Hurvitz SA Martin M Symmans WF Jung KH Huang CS Thompson AM. Neoadjuvant trastuzumab, pertuzumab, and chemotherapy versus trastuzumab emtansine plus pertuzumab in patients with HER2-positive breast cancer (KRISTINE): a randomised, open-label, multicentre, phase 3 trial. Lancet Oncol (2018) 19(1):115–26. doi: 10.1016/S1470-2045(17)30716-7 29175149

[121] Saide A Lauritano C Ianora A. Pheophorbide a: state of the art. Mar Drugs (2020) 18(5):257. doi: 10.3390/md18050257 32423035 PMC7281735

[122] Arumugam P Song JM. Quantitative evaluation of ABC transporter-mediated drug resistance based on the determination of the anticancer activity of camptothecin against breast cancer stem cells using TIRF. IntegrBiol (Camb) (2016) 8(6):704–11. doi: 10.1039/C6IB00021E 27182942

[123] Badrinath N Yoo SY. Recent advances in cancer stem cell-targeted immunotherapy. Cancers (Basel) (2019) 11(3):310. doi: 10.3390/cancers11030310 30841635 PMC6468501

[124] Teitz-Tennenbaum S Wicha MS Chang AE Li Q. Targeting cancer stem cells via dendritic-cell vaccination. Oncoimmunology. (2012) 1(8):1401–3. doi: 10.4161/onci.21026 PMC3518516 23243607

[125] Yamada R Takahashi A Torigoe T Morita R Tamura Y Kanaseki T. Preferential expression of cancer/testis genes in cancer stem-like cells: proposal of a novel sub-category, cancer/testis/stem gene. Tissue Antigens (2013) 81(6):428–34. doi: 10.1111/tan.12113 23574628

[126] Kwapisz D. Pembrolizumab and atezolizumab in triple-negative breast cancer. Cancer Immunol Immunother (2021) 70(3):607–17. doi: 10.1007/s00262-020-02736-z PMC10992894 33015734

[127] Mahmoud AM. Cancer testis antigens as immunogenic and oncogenic targets in breast cancer. Immunotherapy. (2018) 10(9):769–78. doi: 10.2217/imt-2017-0179 PMC6462849 29926750

[128] Yang L Shi P Zhao G Xu J Peng W Zhang J. Targeting cancer stem cell pathways for cancer therapy. Signal transduction targeted Ther (2020) 5(1):1–35. doi: 10.1038/s41392-020-0110-5 PMC7005297 32296030

[129] Zeng X Liu C Yao J Wan H Wan G Li Y. Breast cancer stem cells, heterogeneity, targeting therapies and therapeutic implications. Pharmacol Res (2021) 163:105320. doi: 10.1016/j.phrs.2020.105320 33271295

[130] Quaglino E Conti L Cavallo F. Breast cancer stem cell antigens as targets for immunotherapy. In Seminars in immunology. Academic Press: Elsevier (2020). 47, 101386. 31932198 10.1016/j.smim.2020.101386

